# Modulation of Biofilm Exopolysaccharides by the *Streptococcus mutans vicX* Gene

**DOI:** 10.3389/fmicb.2015.01432

**Published:** 2015-12-21

**Authors:** Lei Lei, Yingming Yang, Mengying Mao, Hong Li, Meng Li, Yan Yang, Jiaxin Yin, Tao Hu

**Affiliations:** ^1^State Key Laboratory of Oral Diseases, Department of Operative Dentistry and Endodontics, West China Hospital of Stomatology, Sichuan UniversityChengdu, China; ^2^Department of Preventive Dentistry, West China Hospital of Stomatology, Sichuan UniversityChengdu, China; ^3^Centre of Infectious Diseases, West China Hospital of Sichuan UniversityChengdu, China

**Keywords:** biofilms, caries prevention, glucosyltransferase, polysaccharides, *Streptococcus mutans*, two component *VicRK* system

## Abstract

The cariogenic pathogen *Streptococcus mutans* effectively utilizes dietary sucrose for the synthesis of exopolysaccharide, which act as a scaffold for its biofilm, thus contributing to its pathogenicity, environmental stress tolerance, and antimicrobial resistance. The two-component system *VicRK* of *S. mutans* regulates a group of virulence genes that are associated with biofilm matrix synthesis. Knockout of *vicX* affects biofilm formation, oxidative stress tolerance, and transformation of *S. mutans*. However, little is known regarding the *vicX*-modulated structural characteristics of the exopolysaccharides underlying the biofilm formation and the phenotypes of the *vicX* mutants. Here, we identified the role of *vicX* in the structural characteristics of the exopolysaccharide matrix and biofilm physiology. The *vicX* mutant (SmuvicX) biofilms seemingly exhibited “desertification” with architecturally impaired exopolysaccharide-enmeshed cell clusters, compared with the UA159 strain (*S. mutans* wild type strain). Concomitantly, SmuvicX showed a decrease in water-insoluble glucan (WIG) synthesis and in WIG/water-soluble glucan (WSG) ratio. Gel permeation chromatography (GPC) showed that the WIG isolated from the SmuvicX biofilms had a much lower molecular weight compared with the UA159 strain indicating differences in polysaccharide chain lengths. A monosaccharide composition analysis demonstrated the importance of the *vicX* gene in the glucose metabolism. We performed metabolite profiling via ^1^H nuclear magnetic resonance spectroscopy, which showed that several chemical shifts were absent in both WSG and WIG of SmuvicX biofilms compared with the UA159 strain. Thus, the modulation of structural characteristics of exopolysaccharide by *vicX* provides new insights into the interaction between the exopolysaccharide structure, gene functions, and cariogenicity. Our results suggest that *vicX* gene modulates the structural characteristics of exopolysaccharide associated with cariogenicity, which may be explored as a potential target that contributes to dental caries management. Furthermore, the methods used to purify the EPS of *S. mutans* biofilms and to analyze multiple aspects of its structure (GPC, gas chromatography-mass spectrometry, and ^1^H nuclear magnetic resonance spectroscopy) may be useful approaches to determine the roles of other virulence genes for dental caries prevention.

## Introduction

Dental plaque as a microbial biofilm is defined as the multi-species community of micro-organisms formed on tooth surfaces (Kroes et al., 1999). Dental caries are promoted by environmental conditions (e.g., changes in pH) that cause ecological shifts favoring the proliferation of aciduric bacteria (Kazor et al., 2003). *Streptococcus mutans* (*S. mutans*) is among the few species that have been consistently linked with caries formation, with the ability to synthesize extracellular polysaccharides that promote formation of the plaque biofilm, and contributes to the pathogenicity of the species (Yamashita et al., 1993; Xiao and Koo, 2010). Exopolysaccharides, which are crucial components of the protective shelter in cariogenic oral biofilms, are recognized as important virulence factors involved in the pathogenesis of dental caries (Bowen and Koo, 2011). Glucans, including water-insoluble glucan (WIG) and water-soluble glucan (WSG), undergo structural modifications resulting from the effects of glucosyltransferases (Gtfs) and fructosyltransferase (Ftf; Rolla et al., 1983), together with dextranase (Dex), a type of glucanase that participates in the degradation of exopolysaccharide (Khalikova et al., 2003), during glucan synthesis. *S. mutans* produces three kinds of Gtfs: GtfB, which synthesizes mostly insoluble glucan (α1,3-linked); GtfD, which synthesizes soluble glucan (α1,6-linked); and GtfC, which synthesizes a mixture of insoluble and soluble glucans (Loesche, 1986). Insoluble glucan promotes the accumulation and binding of microorganisms to the tooth surface (Cross et al., 2007). Fructosyltransferase (Ftf) synthesizes β(2,1)/β(2,6)-linked fructans which are used as a carbohydrate reservoir and may also enhance bacterial adhesion (Rozen et al., 2004). In contrast, Dex degrades glucans by hydrolyzing the glycosidic bonds and additionally inhibits Gtf activity (Khalikova et al., 2003).

The *VicRK* system which was originally identified in *Bacillus subtilis* (*B. subtilis*) is highly conserved and specific to low G+C Gram-positive bacteria (Fukuchi et al., 2000). The *vic* operon comprises three regulatory elements: *vicR*, a response regulator; *vicK*, its cognate histidine kinase; and *vicX*, a putative hydrolase that shares 55% identity to the predicted product (YycJ) in the *B. subtilis* genome (Wagner et al., 2002). In *B. subtilis*, YycJ is a putative hydrolase belonging to the metallo-β-lactamase super family (Fabret and Hoch, 1998). The cellular roles of YycJ have been identified in colony morphology (Szurmant et al., 2007) and cell wall metabolism of *B. subtilis* (Ng et al., 2003). Prior investigations have attempted to demonstrate that the *VicRK* two-component signal transduction system (TCSTS) regulates *gtfB/C/D* and *ftf* genes, which interact in concert to sense and adapt to environmental changes in *S. mutans* (Senadheera et al., 2005; Duque et al., 2011). The genes in the same operon seem to participate in relatively static complexes and lead to a strongly coordinated expression of a set of genes (Bratlie et al., 2010). From this point of view, VicX and the VicR response regulator might interact to regulate important physiological factors in *S. mutans* (Senadheera et al., 2007). The *vicX* gene is the third gene of the operon and may have a signal transduction function in the *VicRK* system in *S. mutans* (Senadheera et al., 2007). It has been documented that deletion of the coding region of the *vicX* gene affects the biofilm formation, sucrose-dependent adhesion, oxidative stress tolerance, and genetic competence (Senadheera et al., 2007). However, the structural characteristics of exopolysaccharide and the expression of virulence factors that may be regulated by *vicX* gene have received limited attention.

The presence of dietary carbohydrates, exopolysaccharide synthesis, and a group of virulence factors modulate the dental biofilm structural characteristics (Raghavan and Groisman, 2010). Despite the importance of exopolysaccharide in bacterial cariogenicity, how the *vicX* gene modulates the biofilm exopolysaccharide characteristics has not yet been elucidated well. Although there are many protocols for the extraction of total bacterial polysaccharides in the planktonic growth, only a few methods have attempted to fractionate and analyze the backbone glucans from biofilms (Seo et al., 2011; Bales et al., 2013).

Considering the crucial role of exopolysaccharide in bacterial cariogenicity, we combined parts of the metabolic glycomics protocols (with modifications) to establish an effective method for purifying exopolysaccharide isolated from *S. mutans* biofilms (Cerca et al., 2005; Comte et al., 2007). The exopolysaccharide matrix constituents, particularly WIG, may affect the dynamic arrangement of biofilm substances, thus helping protect bacterial cells from environmental stress (Stewart and Franklin, 2008). However, the knowledge regarding how exopolysaccharide are three-dimensionally assembled and their association with cariogenicity is limited, especially due to difficulties in studying the structural characteristics of the exopolysaccharide matrix in intact biofilms. In the present study, we focused on the ecological role of *vicX* gene in the structural characteristics of exopolysaccharide that are associated with cariogenicity. To achieve these goals, a previously described *vicX*-deficient strain was recreated and biofilm physiology was investigated (Senadheera et al., 2007). Furthermore, the structural characteristics of the exopolysaccharide isolated from *S. mutans* were determined using the anthrone method for glucan quantification, Gel permeation chromatography (GPC) for molecular weight assessment, acetylation for monosaccharide composition analysis, and ^1^H nuclear magnetic resonance (^1^H NMR) spectroscopy. We focused on the structural characteristics and crucial monosaccharide composition of the exopolysaccharide matrix modulated by *vicX* gene. These studies will ultimately contribute to the management of dental caries by identifying therapeutically important targets.

## Materials and methods

### Bacterial strains, construction of mutants

The *S. mutans* UA159 strain was provided by the State Key Laboratory of Oral Diseases (Sichuan University, Chengdu, China) and the *vicX* deficient strain (SmuvicX) was constructed using the approach as previously described (Senadheera et al., 2007). The accuracy of the deletion was verified using PCR and DNA sequencing (Figure S1). Briefly, an erythromycin resistance cassette was used to disrupt the *vicX* gene region in the *S. mutans* UA159 chromosome by PCR ligation mutagenesis as previously described (Lau et al., 2002; Morrison and Cvitkovitch, 2002). The erythromycin resistance cassette was used as template for amplifying the erythromycin resistance gene (erm^r^) as previously described (Claverys et al., 1995). First, the genomic DNA was extracted from the *S. mutans* UA159. PCR primer pairs VicX-P1/VicX-P2 and VicX-P3/VicX-P4 were used to amplify the flanking regions from genomic DNA, while primers erm-PF and erm-PB were used to amplify the erm^r^ gene. VicX-P1/VicX-P2 were used to amplify the 5′ region flanking the target gene, while VicX-P3/VicX-P4 were used to amplify the 3′ region flanking the target gene. Then purified PCR products of the VicX-P1/VicX-P2 were digested with endonucleases AscI, and PCR product of VicX-P3/VicX-P4 was digested with FseI respectively (New England BioLabs, Beverly, MA, USA). Purified PCR products of the amplified ErmR fragments were digested with the restriction endonucleases AscI and FseI. The primers utilized for construction of the mutagenic construct were listed in Table 1. The ligation was performed by mixing the three digested PCR products before genetic transformation. For the genetic transformation, the overnight *S. mutans* UA159 cultures were diluted 20-fold, grown to an OD_600 nm_ of 0.4. The *S. mutans* competence stimulating peptide (CSP) was added to the culture to achieve a final concentration of 1 μg/mL. Transforming DNA from the ligation reaction was simultaneously added to the culture and incubation was continued as above for 60–90 min (Senadheera et al., 2007). The *vicX*-deficient strains were isolated in BHI plates which contained erythromycin (10 μg/mL). To confirm that integration of the erythromycin gene into the *vicX* coding region, chromosomal DNA was isolated from the mutant strain and PCR amplified using combinations of primers (vicX-P1 and vicX-P4, vicX-P1 and Erm cst-F, and vicX-P4 and Erm cst-B; Senadheera et al., 2007). To do this, chromosomal DNA was isolated with a DNeasy tissue kit (QIAGEN, Valencia, CA, USA). For *vicX* overexpression strains (SmuvicX+), *vicX* gene plus its promoter region were directly cloned into the pDL278 shuttle vector (Bitoun et al., 2011). The SmuvicX+ strains were isolated from Brain Heart Infusion broth (BHI; Difco, Sparks, MD, USA) plates containing 10 μg/mL spectinomycin.

**Table 1:** **Primers for RT-PCR**.

**Primer**	**Nucleotide sequence**	**Amplicon size (bp)**
dexA-F	5′-AGGGCTGACTGCTTCTGGAGT-3′	142
dexA-R	5′-AGTGCCAAGACTGACGCTTTG-3′	
dexB-F	5′-AGAACACCTATGCAATGGGATGCTT-3′	160
dexB-R	5′-GTTGCTGAATGAGTTGTTGATAGGT-3′	
gtfB-F	5′-ACACTTTCGGGTGGCTTG-3′	127
gtfB-R	5′-GCTTAGATGTCACTTCGGTTG-3′	
gtfC-F	5′-CCAAAATGGTATTATGGCTGTCG-3′	136
gtfC-R	5′-TGAGTCTCTATCAAAGTAACGCAG-3′	
gtfD-F	5′-AATGAAATTCGCAGCGGACTTGAG-3′	245
gtfD-R	5′-TTAGCCTGACGCATGTCTTCATTGTA-3′	
ftf-F	5′-ATTGGCGAACGGCGACTTACTC-3′	103
ftf-R	5′-CCTGCGACTTCATTACGATTGGTC-3′	
*vicR*-F	5′-CGCAGTGGCTGAGGAAAATG-3′	157
*vicR*-R	5′-ACCTGTGTGTGTCGCTAAGTGATG-3′	
*vicK*-F	5′-CACTTTACGCATTCGTTTTGCC-3′	102
*vicK*-R	5′-CGTTCTTCTTTTTCCTGTTCGGTC-3′	
*vicX*-F	5′-TGCTCAACCACAGTTTTACCG-3′	127
*vicX*-R	5′-GGACTCAATCAGATAACCATCAGC-3′	
gyrA-F	5′-ATTGTTGCTCGGGCTCTTCCAG-3′	105
gyrA-R	5′-ATGCGGCTTGTCAGGAGTAACC-3′	
vicX-P1	5′-CCAGATTTTTCTTCACCCTTAC-3′	478
vicX-P2	5′-GGCGCGCCTGATACCTCGCCAGATACTG-3′	478
vicX-P3	5′-GGCCGGCCCACTTTTCGGTCTATTTCTGC-3′	401
vicX-P4	5′-AGGCTTGGGTATTCCTAAG-3′	401
erm-PF	5′-GGCGCGCCCCGGGCCCAAAATTTGTTTGAT-3′	876
erm-PR	5′-GGCCGGCCAGTCGGCAGCGACTCATAGAAT-3′	876

### Biofilm growth condition

The wild type UA159, SmuvicX and SmuvicX+ were cultured in BHI without antibiotics overnight at 37°C anaerobically (90% N_2_, 5% CO_2_, 5% H_2_). The overnight cultures of *S. mutans* were further diluted 1:20 in BHI containing 1% sucrose and no antibiotics. Bacterial growth was monitored by measurement of the optical density of the cell culture at 600 nm (OD_600 nm_). The cell culture was adjusted to an OD_600 nm_ of 0.1 in 2 mL of BHI containing 1% sucrose and no antibiotics. The cultures of different stains were inoculated on the glass cover slips in a 24-well cell culture plate and biofilm biomass was quantified after 24 h of growth, respectively.

### RNA isolation, purification, and reverse transcription

After biofilms were established for 24 h, the biofilm samples were harvested by scraping. Cells were collected by centrifugation (4500 rpm, 2422 × g) and RNA was immediately stabilized using the RNAprotect Bacteria Reagent (QIAGEN, Valencia, CA, USA). Cells were then re-suspended in 100 ml of lysis buffer (30 mg/ml lysozyme, [pH 8.0]) and incubated at 37°C with gentle agitation for 30 min. The lysate was further collected and sonicated on ice for two cycles of ultrasonication for 60 s as described previously (Xu et al., 2012). Briefly, 350 μL of chloroform and isoamyl alcohol (24:1) were added and shaken vigorously. The upper layer was transferred to a fresh micro tube. Then, 350 μL high salt solution (0.8 M sodium citrate and 1.2 M sodium chloride) and 250 μL isopropanol was added to precipitate the RNA. The pellet of RNA was washed with 70% alcohol and air-dried. The purity (A260/A280) and concentrations of RNA was assessed by NanoDrop2000 spectrophotometry (Thermo Scientific, Waltham, MA, USA). Total RNA were purified in the RNeasy purification kit (QIAGEN, Valencia, CA, USA) and RNA reverse transcription was performed with QuantiNova reverse transcription kit (QIAGEN, Valencia, CA, USA) in accordance with the recommendations of the supplier.

### Transcription analysis by real-time PCR

Real-time PCR was used to assess *vicR/K/X, gtfB/C/D, ftf*, *gbpB*, and *dexA* mRNA expression levels, using *gyrA* as an internal control. It was conducted as described by the manufacturer in a Bio-Rad CFX96 TM Real-time System (Bio-Rad Laboratories, Hercules, CA, USA) and using a Quantitect SYBR-Green PCR kit (QIAGEN, Valencia, CA, USA). All primers for real-time PCR were obtained commercially (Sangon Biotech, Shanghai, China) and are listed in Table 1. For each real-time PCR, 20 μL of a mixture containing 10 μL of SYBR Premix Ex TaqII, 2.0 μL of template, 0.4 μL of 20 μM PCR Forward Primer, 0.4 μL of 20 μM PCR Reverse Primer, 0.4 μL of ROX Reference Dye and 6.8 μL of deionized water was placed in each well. Real-time PCR was performed as follows: 95°C for 30 s, followed by 40 cycles of 95°C for 5 s and 56°C for 30 s, and 65°C for 5 s. The quantification cycle (Cq) for each qPCR reaction was calculated and the normalized expression of each gene was quantified according to previous studies (Bustin et al., 2009; Zheng et al., 2013). At least a two-fold change was considered a significant difference in gene expression.

### Biofilm assessment and structural imaging

For scanning electron microscopy (SEM), the *S. mutans* biofilms of different strains (UA159, SmuvicX, SmuvicX+) were washed twice with PBS and fixed with 2.5% glutaraldehyde for 8 h. Further preparation included serial dehydration through ethanol solutions (30, 50, 70, 95, and 100%), critical-point drying with liquid CO_2_, and coating with gold for imaging. Scanning electron micrographs of *S. mutans* biofilms of different strains (UA159, SmuvicX, SmuvicX+) were obtained with a scanning electron microscope (Inspect Hillsboro, OR, USA). Specimens were observed at × 2000, × 5000, and × 20,000 magnifications, respectively. For biomass determination, the bacterial cells of the biofilms were labeled with SYTO9 (Invitrogen, Carlsbad, CA, USA) and the exopolysaccharide matrix was stained with an Alexa Fluor 647-labeled dextran conjugate (Invitrogen, Eugene, OR, USA; Yang et al., 2013). Microscopic observation was performed using a laser scanning confocal microscope (CLSM, TSP SP2; Leica, Solms, Germany) with a 63 × oil-immersion objective lens, and biomass quantification was performed via COMSTAT analysis (Xiao et al., 2012). A three-dimensional reconstruction of the biofilms was analyzed using Imaris 7.0.0 software (Bitplane, Zurich, Switzerland). The procedure was repeated three times for three randomly selected views of each specimen. For atomic force microscopy (AFM), the *S. mutans* biofilms were rinsed twice with PBS and dried for 2 min in air. All AFM measurements were performed using an SPM-9500J2 (Shimadzu, Tokyo, Japan) in the contact mode (Mei et al., 2009). The surface roughness (Ra) and each adhesion force were calculated. The external surfaces of the biofilms were examined and the surface roughness average (Ra) was calculated (Iijima et al., 2012). For each adhesion force measurement, the bacterial probe was positioned over the center of a bacterium and 10 force cycles were recorded for five different bacterial cells (Ivanov et al., 2011).

### Anthrone method for polysaccharide measurement

The WIG and WSG of biofilms were extracted from the *S. mutans* biofilms as previously described (Koo et al., 2003). Briefly, the bacteria in 24 h biofilms were collected by scraping and vortexing in PBS buffer. The supernatant was separated for WSG measurement using the anthrone method, and the precipitate was obtained by centrifugation. Subsequently, the precipitate was washed twice with 4 mL of 0.4 M NaOH. After centrifugation, 600 μL of anthrone reagent was added to 200 μL of supernatant, for WIG assessment. The mixtures were then heated at 95°C for 6 min. The absorbance of each sample at 625 nm was monitored on a microplate reader (Gene Co., Hong Kong, China). In addition, the standard curves were prepared with the dextran standard with various concentrations. The corresponding polysaccharide concentration was calculated according to the standard curve (Figure S2).

### Isolation and purification of the exopolysaccharide matrix

Exopolysaccharide extraction and purification from *S. mutans* biofilms were performed according to a previous protocol, with modifications (Bales et al., 2013). Briefly, 5 mL of overnight bacterial culture was added to 100 mL of fresh BHI medium containing 1% sucrose in a polystyrene cell culture flask (Corning Incorporated, Corning, New York), in order to provide a large surface for biofilm formation over 24 h. Cell suspensions were then centrifuged (4500 rpm, 2422 × g) for 25 min at 4°C. Both the supernatant and precipitate were collected, centrifuged, dialyzed, and lyophilized for further purification. The supernatant containing WSG was filtered through a 0.45 μm filter (Corning Incorporated, Corning, New York) and dialyzed using a membrane with a 500–1000 Da molecular weight cutoff (MWCO) against distilled water for 24 h at 25°C.

The precipitate that contained WIG was dissolved in 30 mL of a 1 M NaOH solution and incubated at room temperature with gentle shaking (120 rpm) for 2 h to extract exopolysaccharide. The supernatant of the alkaline solution was collected and 20% (w/v) trichloroacetic acid (TCA) was added to precipitate proteins and nucleic acids on ice for 30 min. The solutions were centrifuged (4500 rpm, 2422 × g) for 30 min at 4°C and the supernatant collected for further purification. A three-fold of the volume of 95% (v/v) ethanol was added and the solutions were kept at 4°C overnight. The resulting precipitate was dialyzed (cellulose membrane with a molecular weight cut-off of 500–1000 Da) against distilled water for 24 h at 25°C; the remaining retentate was lyophilized overnight. The lyophilized powder was dissolved in 5 mL of distilled water and purified on an S-200 gel filtration column (GE Healthcare, Amersham, UK). The polysaccharide fractions that were eluted with distilled water were pooled and lyophilized to dryness.

### Molecular weight distribution of polysaccharides

The GPC is a size exclusion chromatography which separates analytes on the basis of molecular size and is often used for the analysis of polymers (Lathe and Ruthven, 1956). The polymers can be characterized by their average molecular weight (Kruppa et al., 2009; Mao et al., 2009; Cuzzi et al., 2014). Determination of the WIG molecular weight was performed on an Agilent Technologies 1200 series HPLC equipped with a gel permeation chromatographic column (7.8 × 300 mm; TSKgel G5000PW,Tosoh Bioscience, Tokyo, Japan) at 40°C. The mobile phase consisting of 0.5% acetic acid was kept at a flow rate of 0.5 mL/min. The polysaccharide samples were dissolved in distilled water to reach a final concentration of 8 mg/mL. Column calibration was performed using various standard dextrans (Mw (in g/mol): of 1.0 × 10^3^ g/mol, 3.5 × 10^3^ g/mol, 1.0 × 10^4^ g/mol, 1.0 × 10^5^ g/mol, 1.0 × 10^6^ g/mol; Sigma, San Francisco, CA, US). The corresponding molecular weight of samples was calculated using the Agilent GPC software.

The molecular weight of the WSG was estimated using high performance GPC. The column (Shodex KS-804 and KS-802 column, Japan) was maintained at 40°C; the mobile phase was 0.15 M NaCl and was kept at a flow rate of 0.5 mL/min. The polysaccharide samples were dissolved in distilled water to reach a final concentration of 4 mg/mL and the solutions were filtered through a 0.45 μm filter membrane before injection. The eluate was monitored using a refractive index detector (Shodex RI-101, Japan). The column calibration was performed using the P-series dextran standards (P-5, P-10, P-20, P-50, P-100, P-200, P-400, and P-800). Each exopolysaccharide sample was technically replicated three times. The corresponding molecular weights of samples were calculated according to the standard curve (Figure S3).

### Monosaccharide composition analysis of polysacchrides

The monosaccharide composition analysis was performed using the acetylation method (Fujii et al., 2012). Four milligrams of the purified WIG samples were dissolved in 2 M trifluoroacetic acid and hydrolyzed by heating at 99°C for 5 h to dryness. Subsequently, 0.5 mL of 4% sodium borohydride was mixed with the solutions for 1.5 h at ambient temperature. To digest the excess sodium borohydride, acetic acid was added and the solutions were vacuumized. Subsequently, to produce acetylated forms, 1 mL of pyridine and 1 mL of acetic acid anhydride were added and mixed at 95°C for 1 h. Regarding the WSG samples, 2 mg of these were dissolved in 1 mL of 2 M trifluoroacetic acid and hydrolyzed by heating at 100°C for 5 h to dryness. Then, 2 mL of 4% sodium borohydride was mixed with the solution for 2 h at ambient temperature; 1 mL of acetic acid was added to neutralize the excess sodium borohydride, and the solutions were vacuumized. Subsequently, 1 mL of acetic acid anhydride was added, for acetylation, and ultrapure water was added to hydrolyze the excess acetic acid anhydride.

After acetylation, 5 mL of ultrapure water was added to hydrolyze the excess acetic acid anhydride. The solutions were vacuumized to ambient temperature and dissolved in chloroform. The monosaccharide analysis was performed via GC/MS (Gas Chromatography-Mass Spectrometry, Agilent, 19091J-413) using a chromatographic column (HP-5, Agilent) and nitrogen as carrier (injector temperature, 250°C; detector temperature, 250°C). Each exopolysaccharide sample was technically replicated three times. The GC/MS chromatogram demonstrated that the standard monosaccharides could be well separated and identified (Figures S4, S5).

### ^1^H NMR spectroscopy

For the NMR analyses, 20 mg of each polysaccharide sample was lyophilized in 0.5 mL of 99.96% D_2_O (Mei et al., 2015). The ^1^H NMR spectra were recorded on a Bruker AVANCEIII 600 MHz spectrometer (Bruker, Rheinstetten, Germany) and the original chemical shifts of dextran standards determined (Figure S6).

### Animal study

Animal experiments were approved by the Ethics Committee of West China Hospital of Stomatology (Number of permit: SCXK-2009-09). For the animal experiments, caries-susceptible, Osborne-Mendel rats (IVC Experimental Animal Center of Public Health, Sichuan University, Chengdu, China) were used to investigate the effects of the UA159 and *vicX* mutations (SmuvicX) on the formation of dental caries *in vivo* and maintained under specified pathogen-free (SPF) conditions. There were three experimental groups: a blank as the negative control, UA159 as the positive control, and SmuvicX, in which each group comprised 20 test animals. On days 23–30, each rat was infected orally with 200 μL of bacterial suspension that contained the UA159 strain or the SmuvicX strain. The animals were sacrificed on day 50. The lower jaws were divided into right and left pieces. After washing with phosphate-buffered saline twice, two pieces were selected. The lower jaws were dissected and immersed in10% buffered formalin phosphate for 72 h. The rat teeth were observed under a stereomicroscope to determine the caries levels according to modified Keyes score (Keyes, 1958; Koo et al., 1999; Su et al., 2014).

### Statistical analyses

Statistical analyses of the data were performed using SPSS 16.0 (SPSS Inc., Chicago, IL, U.S.). The Shapiro–Wilk test demonstrated whether the data were normally distributed, and Bartlett's test was used to assess the homogeneity of variances. For parametric testing, One-way ANOVA was used to assess statistical significance. For nonparametric testing, the Kruskal–Wallis test and least significant difference (LSD) multiple comparisons were used. The differences of the means of data were considered significant if the *P* < 0.05.

## Results

### Dynamics in the expression of exopolysaccharide associated genes in the Smuvicx influenced synthesis

**Figure 1:**
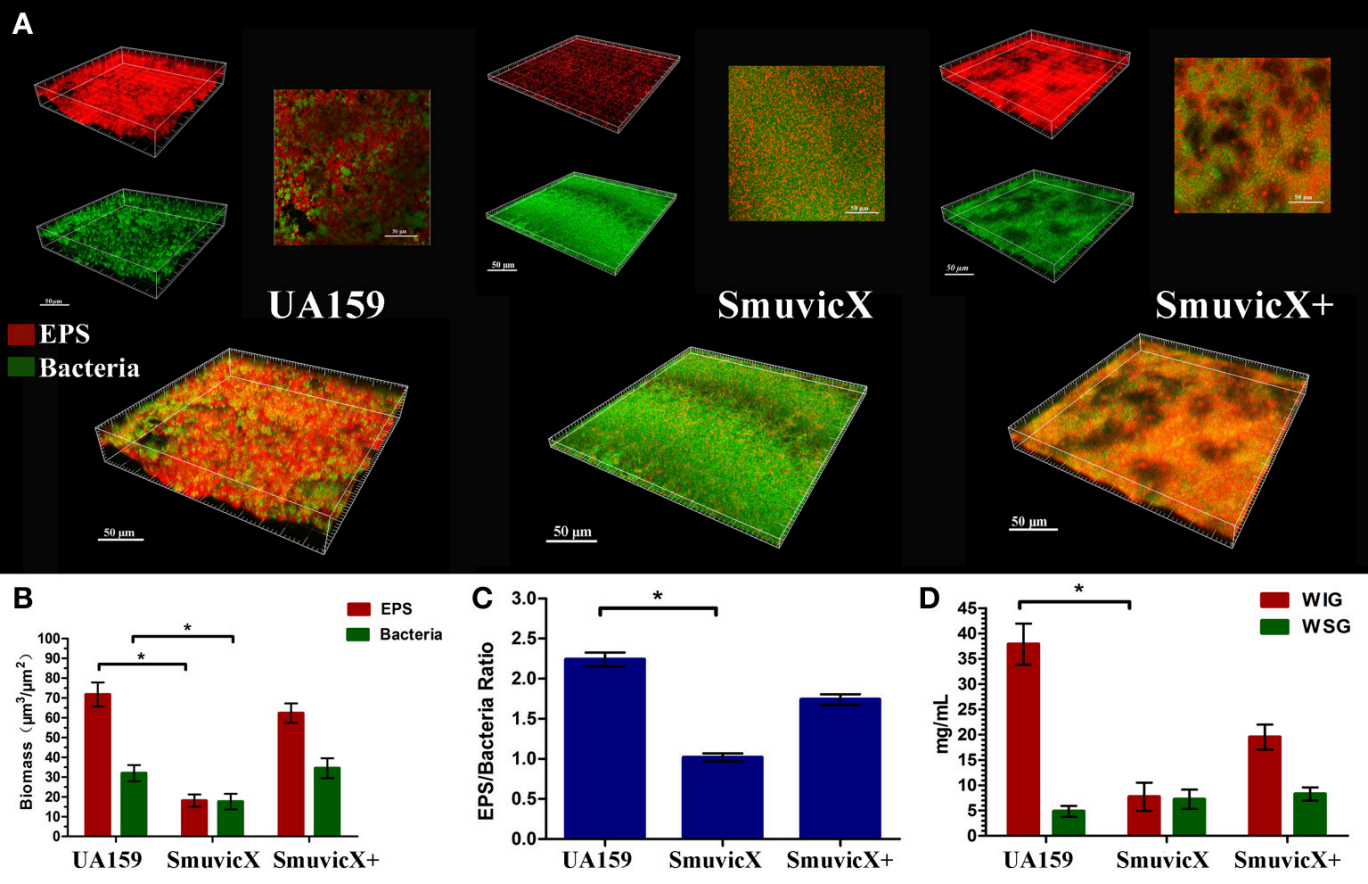
**Laser confocal microscopy of the exopolysaccharide matrix in the biofilm architecture and glucan quantification**. **(A)** Double labeling of the biofilms in the three types of strains. Images were taken at 63× magnification. Green, total bacteria (SYTO 9); red, exopolysaccharide (EPS; Alexa Fluor 647); scale bars, 50 μm. The three-dimensional reconstruction of the biofilms was performed using Imaris 7.0.0. **(B)** Quantitative data of bacterial and exopolysaccharide (EPS) biomass in the three types of strains. Results were averaged from 10 independent cultures of different strains (UA159, SmuvicX, SmuvicX+) and experiments were performed in triplicate. The data are presented as mean ± standard errors. Shapiro–Wilk tests and Bartlett's tests showed that the data were parametric. One-way ANOVA were used to detect the significant effects of variables, ^*^P < 0.05. **(C)** Volume ratio of the exopolysaccharide (EPS) matrix to the bacterial biomass in the biofilms of the three types of strains. The SmuvicX strains exhibited the lowest EPS/bacteria ratio, whereas the UA159 strain had the highest ratio. Results were averaged from 10 independent cultures of different strains (UA159, SmuvicX, SmuvicX+) and the data are presented as mean ± standard errors, ^*^P < 0.05. **(D)** Water-insoluble glucan (WIG) and water-soluble glucan (WSG) of samples from different strains, as measured using the anthrone method. WIG synthesis in the three types of strains was calculated; the UA159 strain exhibited the highest amount of WIG (37.9 ± 4.1), whereas the SmuvicX strains exhibited the lowest amount of WIG (7.7 ± 2.8). Results were averaged from 10 independent cultures of different strains (UA159, SmuvicX, SmuvicX+) and experiments were performed in triplicate. The data are presented as mean ± standard errors. Shapiro–Wilk tests and Bartlett's tests showed that the data were nonparametric. Significant differences were determined using the Kruskal–Wallis test and least significant difference (LSD) multiple comparisons method, ^*^P < 0.05.

**Figure 2:**
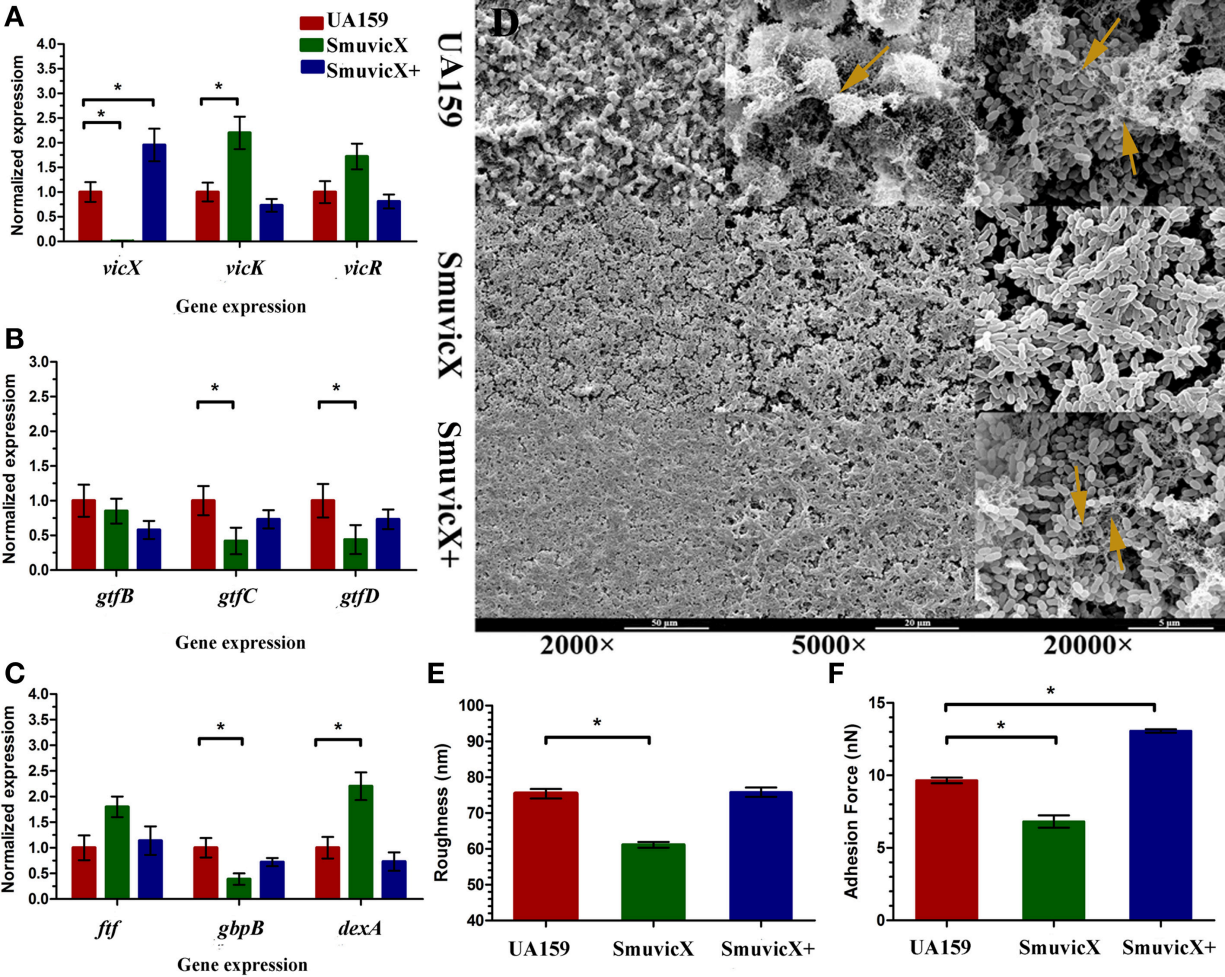
**Dynamics of the expression of exopolysaccharide-associated genes and phenotypic characteristics of ***S. mutans*****. **(A)** Differences in *vicR/K/X* expression in interaction with the *vicRK* transduction system among three types of strains. **(B)** Differences between *gtfB*/*C/D* expressions in exopolysaccharide synthesis of three types of strains. **(C)** Differences in *ftf*, *gbpB*, and *dexA* expression in exopolysaccharide synthetic catalysis and degradation among three types of strains. *S. mutans* gene expression was relatively quantified by real-time PCR using *gyrA* as an internal control and calculated based on the UA159 expression, which was set as 1.0. Results were averaged from 10 independent cultures of different strains (UA159, SmuvicX, SmuvicX+) and experiments were performed in triplicate. The data are presented as mean ± standard deviations. Shapiro–Wilk tests and Bartlett's tests showed that the data were nonparametric. The Kruskal–Wallis test and least significant difference (LSD) multiple comparisons method were used to compare the effects of variables. Asterisks indicate the significant differences among the expressions of genes were considered differentially at a minimal ratio of two-fold changes. **(D)** Scanning electron microscopy (SEM) observation of the architecture of *S. mutans* biofilms. Images were taken at 2000×, 5000×, and 20,000× magnifications, respectively. Clusters of bacterial cells surrounded by enriched exopolysaccharide matrix in the biofilm of the UA159 and SmuvicX+ strains (yellow arrows). The SmuvicX strain seemed to be devoid of exopolysaccharide matrix in the biofilms. Representative images are shown from three randomly selected areas from each sample. **(E)** The means of surface roughness average (Ra) of *S. mutans* biofilms obtained from atomic force microscopy (AFM) experiments were calculated. The values obtained for SmuvicX (61.11 ± 2.55) were lower than those obtained for the UA159 (75.4 ± 4.23) and SmuvicX+ (75.81 ± 4.1) strains. Results were averaged from 10 independent cultures of different strains (UA159, SmuvicX, SmuvicX+) and experiments were performed in triplicate. The data are presented as mean ± standard errors. Shapiro–Wilk tests and Bartlett's tests showed that the data were parametric. One-way ANOVA were used to detect the significant effects of variables, ^*^P < 0.05. **(F)** The values of adhesion force data were obtained from AFM. The SmuvicX+ strain showed the highest average force (13.1 ± 0.64), whereas the SmuvicX strain exhibited the lowest force of adhesion (6.8 ± 2.3); Results were averaged from 10 independent cultures of different strains (UA159, SmuvicX, SmuvicX+) and experiments were performed in triplicate. The data are presented as mean ± standard errors. Shapiro–Wilk tests and Bartlett's tests showed that the data were nonparametric. Significant differences were determined using the Kruskal–Wallis test and LSD multiple comparisons method, ^*^P < 0.05.

By double staining for CLSM, we found that the knockout of *vicX* gene markedly decreased the production of exopolysaccharide, and the biofilms seemed to be thin and were unable to form microcolonies (Figure 1A). This finding was further confirmed by quantitative analysis of biomass of bacteria and exopolysaccharide of the *S. mutans* strains (Figure 1B). Furthermore, the Smu*vicX* exhibited the lowest exopolysaccharide/bacteria volume ratio, which indicated the essential role of *vicX* gene in the exopolysaccharide architecture development (Figure 1C). Concomitantly, the amount of WIG and WSG in *S. mutans* that was obtained using the anthrone method indicated that the deletion of *vicX* gene inhibited WIG synthesis (Figure 1D). WIG synthesis in the UA159 strain was the highest (37.9 ± 4.1 mg/mL), whereas SmuvicX was the lowest (7.7 ± 2.8 mg/mL; Figure 1D). The expression profiles of *vicR/K/X, gtfB/C/D, ftf*, *gbpB*, and *dexA* genes are shown in Figure 2. Compared with the UA159 strain, the expression of *vicK, vicR, ftf*, and *dexA* in the SmuvicX was upregulated by 2.1-, 1.7-, 1.8-, and 2.2-folds, respectively (Figures 2A,C). However, the data revealed that the transcriptional levels of *gtfC, gtfD*, and *gbpB* were significantly decreased in SmuvicX (Figures 2B,C).

### Knockout of *vicX* gene suppressed the assembly of biofilms and the architecture of the exopolysaccharide matrix

We examined the morphology of *S. mutans* biofilms on glass cover slides using SEM (Figure 2D). After the introduction of 1% sucrose, a heterogeneous layer of exopolysaccharide formed and the cells were densely packed with an enriched exopolysaccharide matrix in the UA159 and SmuvicX+ strains. However, the SmuvicX seemed to be devoid of exopolysaccharide matrix in the biofilms. The means of the Ra and adhesion force values of *S. mutans* biofilms obtained from AFM were calculated (Figures 2E,F). With a decrease in the exopolysaccharide matrix, the adhesion between the bacterial cells and the AFM probe in the SmuvicX was impaired (6.8 ± 2.3 nN). Also, a decrease in surface roughness (61.1 ± 2.3 nm) was noted in the SmuvicX biofilm compared to the UA159 and SmuvicX+ strains. We verified that knockout of the *vicX* gene led to a lower cariogenic incidence in the rats in a specific pathogen-free (SPF) rat model, according to a modified Keyes' counting method (Table 2). Additionally, we found that inactivating the *vicX* gene may lead to the dysregulation of *S. mutans* cariogenicity, which emphasized that *vicX* may have relevance for the characterization of biofilm formation *in vivo*.

**Table 2:** **Caries unit scores**.

	**Sucal caries**			**Smooth caries**			**Total**
	***D*_E_**	***D*_s_**	***D*_X_**	***D*_E_**	***D*_s_**	***D*_X_**	
UA159	4.84 ± 1.3	3.74 ± 1.1	0.89 ± 0.5	0.8 ± 0.4	0	0	10.26 ± 1.3
SmuvicX	3.7 ± 1.2^*^	1.05 ± 0.6^*^	0	0	0	0	4.95 ± 0.5^*^
Control	2.6 ± 0.8^*^	0.68 ± 0.4^*^	0	0	0	0	3.42 ± 0.5^*^

*The depths of penetration are under three headings: enamel only (D_E_), dentinal (Ds), and extensive dentinal (Dx). The data represent thecarious lesions score according to modified Keyes scores. By using UA159 as a reference, significant differences were determined using the Kruskal–Wallis test and least significant difference (LSD) multiple comparisons method. Asterisks indicate P < 0.05; (n = 20)*.

### Declined WIG amount significantly affected the structure of exopolysaccharide matrix in the *vicX* mutant biofilm

**Figure 3:**
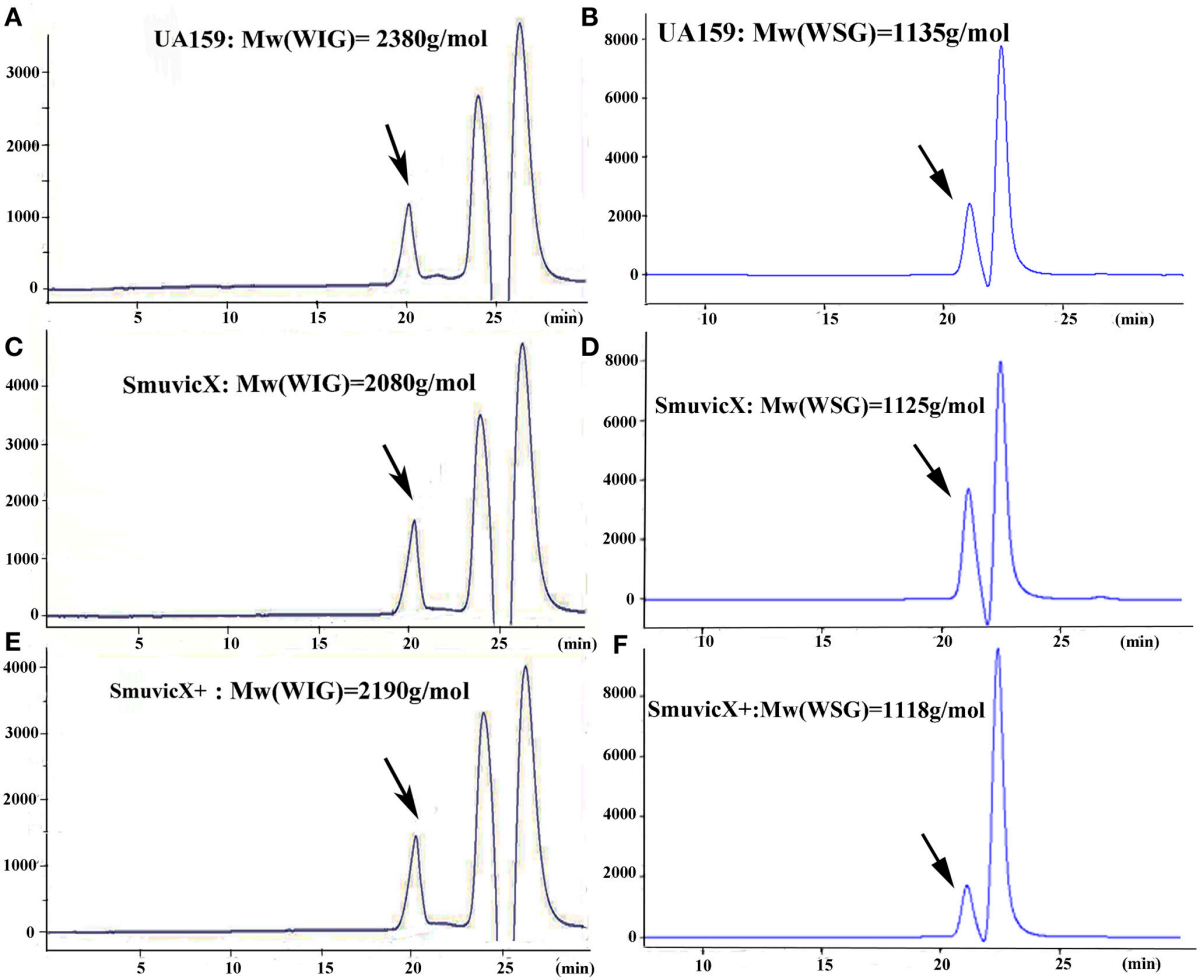
**The molecular weight distribution of the polysaccharides was estimated using high-performance gel permeation chromatography (GPC)**. The GPC analysis demonstrated that each peak from different *S. mutans* strains corresponded to an average molecular weight of 2.38 × 10^3^ g/mol for the water-insoluble glucan (WIG) of the UA159 strain (retention time: 20.12 min; **A**); a molecular weight of 1.14 × 10^3^ g/mol for the water-soluble glucan (WSG) of the UA159 strain (retention time: 20.98 min; **B**); a molecular weight of 2.08 × 10^3^ g/mol for the WIG of the SmuvicX strain (retention time: 20.27 min; **C**); a molecular weight of 1.13 × 10^3^ g/mol for the WSG of the SmuvicX strain (retention time: 21.0 min; **D**); a molecular weight of 2.19 × 10^3^ g/mol for the WIG of the SmuvicX+ strain (retention time: 20.21 min; **E**); and a molecular weight of 1.12 × 10^3^ g/mol for the WSG of the SmuvicX+ strain (retention time: 21.01 min; **F**). Each exopolysaccharide sample was technically replicated three times. The data represent the average molecular weight for the entire polymer distribution.

**Figure 4:**
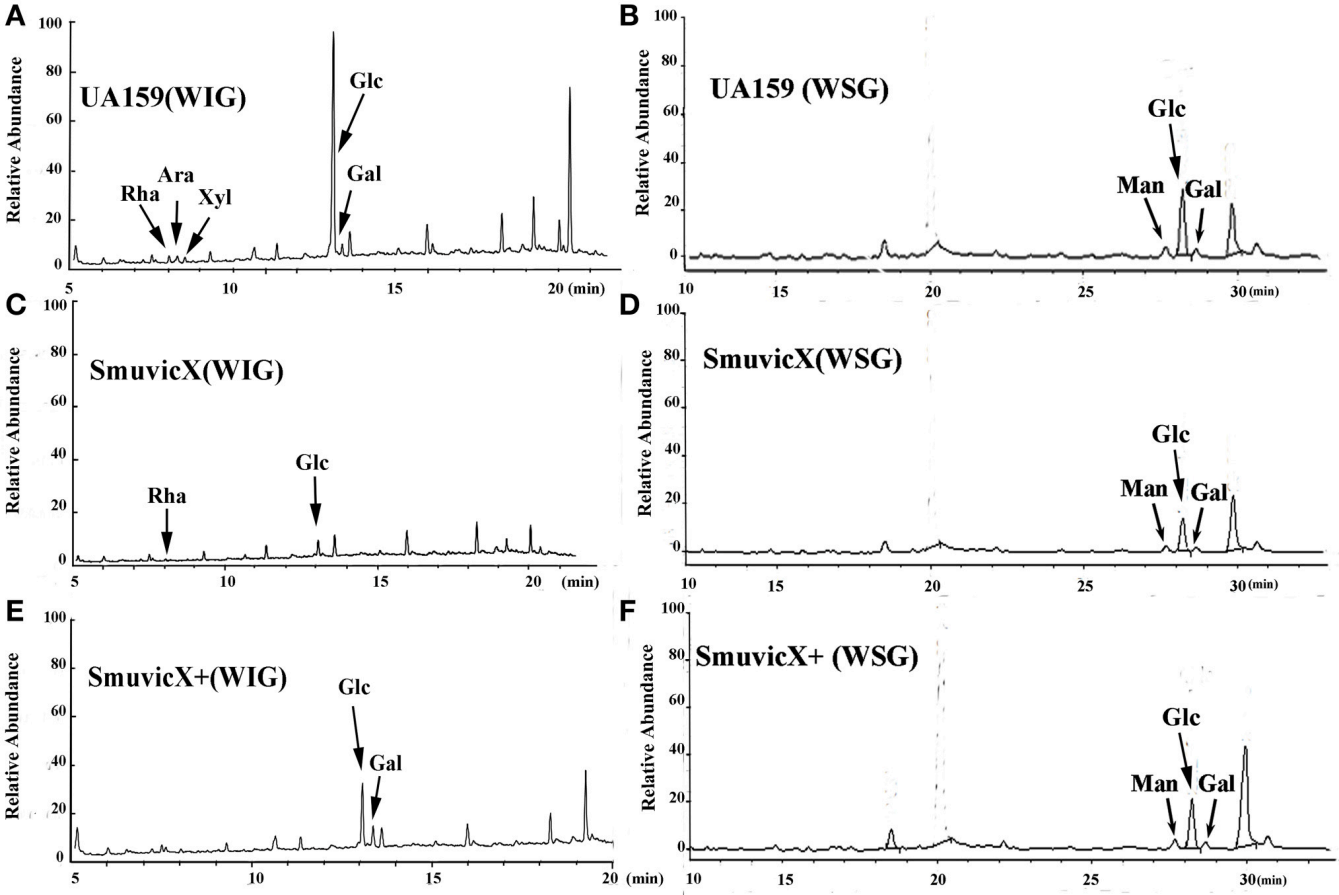
**A monosaccharide composition analysis of the polysaccharide was carried out using a preparation that was precipitated by acetylation**. The GC/MS profile of the monosaccharide composition analysis showed that the water-insoluble glucan (WIG) of the UA159 strain consisted of rhamnose (Rha, retention time: 8.030 min), arabinose (Ara, retention time: 8.273 min), xylose (Xyl, retention time: 8.513 min), glucose (Glc, retention time: 13.061 min), and galactose (Gal, retention time: 13.351 min) with a molar ratio of 1.74:2.4:1.0:58.31:4.19 **(A)**; the water-soluble glucan (WSG) of the UA159 strain contained Man (retention time: 27.690 min), Glc (retention time: 28.225 min), and Gal (retention time: 28.694 min) with a molar ratio of 12.1:70.47:7.27 **(B)**; the WIG of the SmuvicX strain contained Rha (retention time: 8.033 min)and Glc (retention time: 13.069 min) with a molar ratio of 1:13 **(C)**; the WSG of the SmuvicX strain consisted of Man (retention time: 27.690 min), Glc (retention time: 28.223 min), and Gal (retention time: 28.694 min) with a molar ratio of 14.14:53.56:6.24 **(D)**; the WIG of the SmuvicX+ strain contained Glc (retention time: 13.060 min) and Gal (retention time: 13.352 min) with a molar ratio of 2.19:1.0 **(E)**; and the monosaccharide components of the WSG of the SmuvicX+ strain included Man (retention time: 27.690 min), Glc (retention time: 28.238 min), and Gal (retention time: 28.694 min) with a molar ratio of 19.11:95.85:17.75 **(F)**. Each exopolysaccharide sample was technically replicated three times. The data represent the average molar ratio of each monosaccharide.

**Figure 5:**
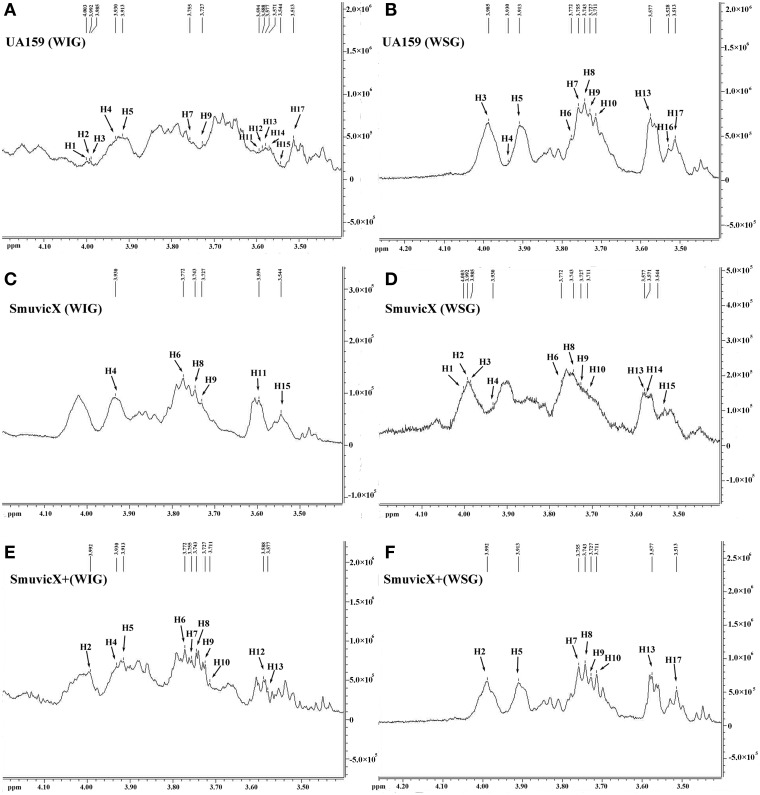
**The structural properties of the glucan of ***S. mutans*** biofilms were further elucidated using 600 MHz ^**1**^H nuclear magnetic resonance (NMR) spectroscopy**. Several sharp well-resolved peaks corresponding to the dextran standard metabolites could be observed in each of the strains. The ^1^H NMR spectrum of the water-insoluble glucan (WIG) of the UA159 strain consisted mostly of signals at 4.003, 3.992, 3.985, 3.9301, 3.913, 3.755, 3.727, 3.594, 3.588, 3.577, 3.571, 3.544, and 3.513 ppm **(A)**; the ^1^H NMR spectrum of the water-soluble glucan (WSG) of the UA159 strain consisted mostly of signals at 3.985, 3.9301, 3.913, 3.772, 3.755, 3.743, 3.727, 3.711, 3.577, 3.528, and 3.513 ppm **(B)**; the ^1^H NMR spectrum of the WIG of the SmuvicX strain consisted of lesser signals at 3.930, 3.772, 3.743, 3.727, 3.594, and 3.544 ppm **(C)**; the ^1^H NMR spectrum of the WSG of the SmuvicX strain consisted of signals at 4.003, 3.992, 3.985, 3.930, 3.772, 3.743, 3.727, 3.711, 3.577, 3.571, and 3.544 ppm **(D)**; the ^1^H NMR spectrum of the WIG of the SmuvicX+ strain consisted of signals at 3.992, 3.930, 3.913, 3.772, 3.755, 3.743, 3.727, 3.711, 3.588, and 3.577 ppm **(E)**; and the ^1^H NMR spectrum of the WSG of the SmuvicX+ strain consisted of signals at 3.992, 3.913, 3.755, 3.743, 3.727, 3.711, 3.571, and 3.544 ppm **(F)**.

Exopolysaccharide matrix formation in the SmuvicX was greatly impaired compared to the UA159 strain, and the few small microcolonies were found to be randomly distributed (Figure 1A). We extracted and purified exopolysaccharide from the three strains in biofilms and identified the role of *vicX* in the structural characteristics of the exopolysaccharide using metabolic glycomics approaches (Figures 3–5). The molecular weight of exopolysaccharide was determined using high performance GPC which revealed that the WSG from *S. mutans* had varying molecular weights (Figures 3B,D). In particular, the WIG isolated from SmuvicX presented a much lower molecular weight (2.08 × 10^3^ g/mol) compared with those from the UA159 strain (2.38 × 10^3^ g/mol), which indicated differences in the length of the polysaccharide chains between the two strains (Figures 3A,C). The SmuVicX+ strain also had a reduced WIG molecular weight (2.19 × 10^3^ g/mol) compared to the UA159 strain (Figures 3A,E). However, the molecular weight of WSG in the SmuVicX+ biofilm was nearly that of the wild-type strain (Figures 3B,F). Subsequently, a monosaccharide composition analysis revealed that the WIG and WSG from *S. mutans* primarily consisted of glucose and variable amounts of galactose and mannose at varying molar ratios (Figure 4). In particular, the results showed that glucose in the WIG of SmuvicX greatly declined and galactose was absent compared to the UA159 strain (Figures 4A,C). Also, the SmuvicX+ strain had a decrease in glucose in the WIG compared to the wild type strain (Figures 4A,E). However, there were no significant differences in molar ratios of the monosaccharide composition among the WSGs of the wide type, SmuvicX and SmuvicX+ strains. The structural properties of the glucan from *S. mutans* were further elucidated by ^1^H NMR spectroscopy (Figure 5). The primary structural analysis in the ^1^H NMR spectra of carbohydrates is based on signals in the range of 3–4.2 ppm (Duus et al., 2000). In this study, the original chemical shifts of exopolysaccharides were expressed within the range of 3.4–4.2 ppm. Although there were several resonances present in the three strains, differences between the strains were visible within the region of 3.4–4.2 ppm (NMR raw data in the Supplementary Materials). Particularly, the ^1^H NMR spectrum showed that, compared with the UA159 strain, several chemical shifts were absent in both the WIG and WSG from the SmuvicX and SmuvicX+ strains (Figure 5).

## Discussion

Our results illustrate the structural characteristics of the exopolysaccharide and the architecture of the biofilm modulated by the *vicX* gene. The exopolysaccharide-rich matrix facilitated the formation of microcolonies within the densely packed biofilm, whereas the structural characteristics of the exopolysaccharide in SmuvicX strain induced “desertification” in the biofilm scaffold. In this context, a heterogeneous layer of exopolysaccharide formed with cells densely packed in an enriched exopolysaccharide matrix in the UA159 and SmuvicX+ strains. However, the SmuvicX biofilm appeared to be devoid of exopolysaccharide matrix in the biofilms and exhibited impaired exopolysaccharide-enmeshed cell clusters. As a result, the *vicX* mutant biofilm was thin and unable to form microcolonies by double staining for CLSM.

### *Vicx* affects *gtfC/D* expression and inhibits the exopolysaccharide matrix architecture

*S. mutans* effectively utilizes dietary sucrose for polysaccharide production and expresses three glucosyltransferases (encoded by *gtfB, gtfC*, and *gtfD*) and a fructosyltransferase (Loesche, 1986). In contrast, a dextranase (DexA) participates in the degradation of glucans (Hayacibara et al., 2004). Our data revealed that the transcriptional levels of *gtfC, gtfD*, and *gbpB* were significantly decreased in the SmuvicX (Figures 2B,C) after 24 h incubation for biofilm formation, suggesting a *vicX*-mediated positive influence on glucan synthesis and initial adherence. On the other hand, the expressions of *vicR, vicK*, and *dexA* were significantly up-regulated in SmuvicX (Figures 2A,C). An increase in expression of *dexA* reduced exopolysaccharide by degradation and glucan synthesis in the SmuvicX strain significantly declined compared to the UA159 strain (Figure 1D).

The results of *gtfB/C* genes expressions in this study appear to be in contrast with the results reported in Senadheera et al. (2007) showing that knockout of *vicX* did not affect levels of *gtfB* and *gtfC* in planktonic condition. The gene expression patterns of *S. mutans* in confluent biofilms are significantly different from planktonic conditions (Arirachakaran et al., 2007). Analysis of transcription levels in *S. mutans* grown in various nutrient media revealed significant alterations in the expression of the *gtfB/C, ftf*, and *vicR* genes involved in biofilm formation (Shemesh et al., 2007). When exopolysaccharides are inhibited, especially WIGs, synthesis of the SmuvicX biofilms is primarily due to the reduced expression of *gtfC/D*. The surface-adsorbed GtfC/D enzyme produces large amounts of glucans and its deletion drastically reduces *S. mutans* adherence to smooth surfaces (Thurnheer et al., 2006). GtfC can catalyze both the insoluble and soluble glucans which may play a more dominant role in biofilm formation (Hanada and Kuramitsu, 1988).

Interestingly, the expressions of *gtfC/D* genes were down-regulated in SmuvicX while *vicR* was significantly up-regulated in this study. However, it has been reported that VicR binds specifically to the promoter regions of *gtfB* and *gtfC*, and VicR positively regulates *gtfB/C/D* (Senadheera et al., 2005; Biswas and Biswas, 2006). Despite this, it is important to notice that VicR and GcrR may co-bind to the promoters of *gtfC/D* and *gbpB* genes identified by electrophoretic motility shift assays (Stipp et al., 2013). These data supported that VicR interacts with GcrR cooperatively coordinate functions of *gtfC/D* and *gbpB* genes involved in cell division and exopolysaccharide synthesis. Additionally, genes that participate in the same operon seem to participate in relatively static complexes (Bratlie et al., 2010), the *vicX* gene, which is part of a tricistronic operon, tricistronic operon, may have a signal transduction function in the *VicRK* system *vicX* mutagenesis may have polar effects on *VicRK* transcription unit, and this may explain the different phenotypes observed here. It would be very interesting to mechanisms involved with the *VicRK* system that co-regulate *gtfBCD* expressions.

### The spatial arrangement between WIG and bacterial cells induced “desertification” in the biofilm scaffold of the *vicX* mutant

The essential role of *vicX* gene in the biofilm development was revealed by the failure to assemble enriched exopolysaccharide-enmeshed microcolonies which induced a “desertification” appearance in the SmuvicX biofilm. The SmuviX biofilms shown in the SEM micrographs obtained at lower magnification seemed “denser” than those of the UA159 strain, which is in agreement with the Senadheera paper (Senadheera et al., 2007). However, the formation of the exopolysaccharide matrix was greatly impaired, as observed at higher magnification (Figure 2D). Hence, it is reasonable to conclude that a lack of exopolysaccharide matrix in the SmuvicX biofilms led to a thin but mostly homogeneous accumulation of bacterial cells (Figure 1A), which may seem “denser” at lower magnification in the biofilms. The deletion of *vicX* gene decreased the biomass of the biofilms as a consequence of the exopolysaccharide matrix reduction (Figure 1B). In addition, altered expression of the *gtfB/C/D, dexA* and *gbpB* were associated with the phenotypic differences of the SmuvicX biofilm. The reduced expression of *gtfC/D* resulted in impaired exopolysaccharide biosynthesis and an increase in expression of *dexA* reduced exopolysaccharide by degradation of the SmuvicX biofilms. The structurally rigid WIG contributed to the scaffolding of the biofilms (Thurnheer et al., 2006) and the lack of WIG suppressed the structural cell-to-matrix bridging, causing the disassembly of the exopolysaccharide matrix and the “desertification” appearance that was detected in the Smu*vicX* biofilm (Figures 1A, 2D). The biofilm roughness may have a positive structural function in the exopolysaccharide matrix, which facilitates the penetration of the nutrients and surface aggregation (Bester et al., 2010). In the present study, down-regulated expression of *gbpB* reduced the SmuvicX biofilm adhesion and attachment to exopolysaccharide matrix. Also, it has been established that the depletion of *gbpB* resulted in altered cell surface properties and sensitivity to osmotic and oxidative stresses (Lynch et al., 2007; Duque et al., 2011). Based on present data, a decrease in *gbpB* expression impaired initial phases of sucrose-dependent biofilm formation. Due to the altered expression of *gtfB/C/D* and *dexA*, a lack of exopolysaccharide matrix likely reduced the roughness and adhesion force in the *vicX* mutant biofilm.

### Structural characteristics of the exopolysaccharide matrix by knockout of vicX gene yield a non-virulent microbial biofilm

It is apparent that the combination of biochemical and metabolic techniques may help elucidate the manner via which the exopolysaccharide matrix is spatially arranged (Bales et al., 2013). The molecular weight of the WIG produced by the UA159 strain is 2380 g/mol, which implies that highly insoluble glucans may be critical for matrix construction (Xiao et al., 2012). Particularly, the SmuvicX strain had a reduced WIG molecular weight compared to the wild type (Figures 3A,C). However, the SmuVicX+ strain with a reduced WIG molecular weight (Figures 3A,E) had a similar biofilm phenotype and exopolysaccharide matrix architecture compared to the UA159 (Figures 1A, 2D) which may indicate that differences in molecular weights of the WIGs do not affect biofilm structure.

The availability of monosaccharide compositions also influenced the exopolysaccharide matrix and it has shown that glucose has become the dominant monosaccharide of the WIG (Figure 4). The glucose metabolism facilitates the conversion of sucrose into acids via the glycolytic pathway (Maehara et al., 2005), with low pH values favoring the growth of aciduric bacteria and the glucan binding of *S. mutans* results in the activation of exopolysaccharide-sheltered matrix aggregation (Lemos and Burne, 2008). The inhibited WIG synthesis of the SmuvicX biofilms was primarily due to the reduced expression of *gtfC/D*. On the other hand, the expression of *dexA* was significantly up-regulated in SmuvicX and reduced exopolysaccharide by degradation. It has been reported that glucans are dispelled by Dex via the glycosidic bonds digestion and Gtf activity also is inhibited in the presence of Dex (Khalikova et al., 2003). Furthermore, the degradation of glucans by DexA in the SmuvicX strain increased the availability of fermentable sugars (Klein et al., 2010). As a result, the metabolism of these carbohydrates may also contribute to the architectural remolding of the exopolysaccharides matrix. However, there were no significant differences in *gtfC/D* or *dexA* expression between SmuvicX+ and wild-type cells. Consistently, these results indicated that the altered expressions of *gtfC/D* and *dexA* contributed to drastically inhibited glucose composition which resulted in phenotypic changes in SmuvicX strain. The SmuvicX biofilms were dynamically shifted toward “desertification,” as they were devoid of enriched exopolysaccharide–microcolony complexes. We found that knockout of the *vicX* gene led to a lower cariogenic incidence in the rats, according to a modified Keyes' counting method (Keyes, 1958; Koo et al., 1999; Su et al., 2014). The SmuvicX strain is associated with a marked reduction in the development of carious lesions which emphasized *vicX* may have relevance characterization for biofilm formation *in vivo* (Table 2). Particularly, SmuvicX mutants exhibited a significantly decreased number of sulcal caries compared with those in the UA159 group *in vivo*. To further elucidate the carbohydrate structures, ^1^H NMR spectroscopy based on highly predictable chemical shifts (Duus et al., 2000) was used to classify the different profiles among the three different strains. Based on the resonances clustered from 3.4 to 4.2 ppm, several chemical shifts were absent in both WSG and WIG from the SmuvicX strain which indicated variability in the carbohydrate composition and linkage of these glucans (Figures 5A–D).

Interestingly, several representative signals from the UA159 strain were not represented in the SmuvicX+ strain (Figures 5E,F). It could be speculated that the introduction of an exogenous plasmid vector carrying *vicX* gene may interfere with the role of the *VicRK* system in regulating intracellular homeostasis in *S. mutans* (Senadheera et al., 2009). As the limited dispersion of chemical shifts of nonanomeric protons in ^1^H NMR spectra was not adequate for the complete determination of the carbohydrates structure, perhaps contemporary NMR studies could utilize 2-D spectra (Toukach and Ananikov, 2013) to investigate the structural complexity of polysaccharides. Although the expression of *gtfBCD* was modestly reduced in the SmuvicX biofilm, the biosynthesis of glucans was drastically inhibited. Further studies are warranted to determine the role of *vicX* gene in the interaction with *VicRK* regulators by examining protein levels of *gtfBCD* and enzymatic activity of each Gtf.

In conclusion, the current study provides structural insights regarding the functional roles of *vicX* gene in exopolysaccharide synthesis, especially in terms of WIG. The biofilm phenotypes that were associated with glucose monosaccharide composition differences and altered expressions of *gtfC/D* shifted the dental biofilm from a virulent to a nonvirulent condition in SmuvicX. Thus, these results indicate the possibility of developing specific inhibitors targeting *vicX* as an effective dental caries prevention method. Novel molecules or commercially available compounds against the crystal structure of the domain of the VicX protein could be identified that are capable of inhibiting exopolysacchride synthesis and biofilm formation in *S. mutans*. Furthermore, the methods used to purify and analyze the exopolysaccharide of *S. mutans* biofilms may be a useful approach to verify the roles of virulent genes for caries prevention.

## Author contributions

LL contributed to conception, design, acquisition, analysis, and interpretation, drafted, and critically revised the manuscript; YMY contributed to conception, design, acquisition, and interpretation, drafted and critically revised the manuscript; MM contributed to analysis and drafted the manuscript; HL contributed to analysis and drafted the manuscript; ML contributed to analysis and drafted the manuscript; YY contributed to acquisition and drafted the manuscript; JY contributed to acquisition and drafted the manuscript; TH contributed to conception, design, and analysis, drafted and critically revised the manuscript. All authors gave final approval and agree to be accountable for all aspects of the work.

### Conflict of interest statement

The authors declare that the research was conducted in the absence of any commercial or financial relationships that could be construed as a potential conflict of interest.

## Supplementary material

Figure S1 **PCR fragment analysis in the ***vicX*** mutant (1) DNA marker; (2) P1–P2 PCR fragment, WT template (478 bp); (3) P3–P4 PCR fragment, WT template (401 bp); (4) erythromycin (erm) cassette PCR confirmation fragment, mutant template (876 bp); (5) P1–PR PCR confirmation fragment, M template (1354 bp); (6) PF–P4 PCR confirmation fragment, mutant template (1279 bp); (7) P1–P4 PCR fragment for confirmation, mutant template (1760 bp); (8) DNA marker**.

Figure S2 **Standard curve for the anthrone method, generated based on known concentrations of dextran**.

Figure S3 **Standard curve of high-performance gel permeation chromatography (GPC), for determination of the molecular weight of the dextran; the linear correlation between the logMw and retention time was calculated (***y*** = −0.369***x*** + 10.80; ***R***^**2**^ = 0.978)**.

Figure S4 **GC/MS chromatogram of six standard monosaccharides for monosaccharide composition analysis of WIG: (1) rhamnose (Rha, retention time: 8.030 min); (2) arabinose (Ara, retention time: 8.270 min); (3) Xylose (Xyl, retention time: 8.514 min); (4) mannose (Man, retention time: 12.956 min); (5) glucose (Glc, retention time: 13.066 min); and (6) galactose (Gal, retention time: 13.346 min)**.

Figure S5 **GC/MS chromatogram of seven standard monosaccharides for monosaccharide composition analysis of WSG: (1) rhamnose (Rha, retention time: 15.951 min); (2) fructose (Fuc, retention time: 16.408 min); (3) arabinose (Ara, retention time: 16.631 min); (4) Xylose (Xyl, retention time: 17.860 min); (5) mannose (Man, retention time: 27.690 min); (6) glucose (Glc, retention time: 28.243min); and (7) galactose (Gal, retention time: 28.694 min)**.

Figure S6 **Several sharp and well-resolved peaks corresponding to the dextran standard metabolites could be observed at 600 MHz ^**1**^H nuclear magnetic resonance (NMR) spectroscopy (0.01% Deuterium generation of trimethyl silane sodium propionate as an internal control)**. The 1D ^1^H NMR spectrum for each of the strains consisted mostly of signals at 4.003 (H1), 3.992 (H2), 3.985 (H3), 3.9301 (H4), 3.913 (H5), 3.772 (H6), 3.755 (H7), 3.743 (H8), 3.727 (H9), 3.711 (H10), 3.594 (H11), 3.588 (H12), 3.577 (H13), 3.571 (H14), 3.544 (H15), 3.528 (H16), and 3.513 (H17) ppm.

## References

[B1] Arirachakaran P. Benjavongkulchai E. Luengpailin S. Ajdić D. Banas J. A. (2007). Manganese affects *Streptococcus mutans* virulence gene expression. Caries Res. 41, 503–511. 10.1159/000110883 17992013 PMC2820327

[B2] Bales P. M. Renke E. M. May S. L. Shen Y. Nelson D. C. (2013). Purification and characterization of biofilm-associated EPS exopolysaccharides from ESKAPE organisms and other pathogens. PLoS ONE 8:e67950. 10.1371/journal.pone.0067950 23805330 PMC3689685

[B3] Bester E. Kroukamp O. Wolfaardt G. M. Boonzaaier L. Liss S. N. (2010). Metabolic differentiation in biofilms as indicated by carbon dioxide production rates. Appl. Environ. Microbiol. 76, 1189–1197. 10.1128/AEM.01719-09 20023078 PMC2820951

[B4] Biswas S. Biswas I. (2006). Regulation of the glucosyltransferase (*gtfBC*) operon by CovR in *Streptococcus mutans*. J. Bacteriol. 188, 988–998. 10.1128/JB.188.3.988-998.2006 16428403 PMC1347363

[B5] Bitoun J. P. Nguyen A. H. Fan Y. Burne R. A. Wen Z. T. (2011). Transcriptional repressor Rex is involved in regulation of oxidative stress response and biofilm formation by *Streptococcus mutans*. FEMS Microbiol. Lett. 320, 110–117. 10.1111/j.1574-6968.2011.02293.x PMC3115380 21521360

[B6] Bowen W. H. Koo H. (2011). Biology of *Streptococcus mutans*-derived glucosyltransferases: role in extracellular matrix formation of cariogenic biofilms. Caries Res. 45, 69–86. 10.1159/000324598 21346355 PMC3068567

[B7] Bratlie M. S. Johansen J. Drabløs F. (2010). Relationship between operon preference and functional properties of persistent genes in bacterial genomes. BMC Genomics 11:71. 10.1186/1471-2164-11-71 20109203 PMC2837039

[B8] Bustin S. A. Benes V. Garson J. A. Hellemans J. Huggett J. Kubista M.. (2009). The MIQE guidelines: minimum information for publication of quantitative real-time PCR experiments. Clin. Chem. 55, 611–622. 10.1373/clinchem.2008.112797 19246619

[B9] Cerca N. Pier G. B. Vilanova M. Oliveira R. Azeredo J. (2005). Quantitative analysis of adhesion and biofilm formation on hydrophilic and hydrophobic surfaces of clinical isolates of *Staphylococcus epidermidis*. Res. Microbiol. 156, 506–514. 10.1016/j.resmic.2005.01.007 15862449 PMC1356821

[B10] Claverys J. P. Dintilhac A. Pestova E. V. Martin B. Morrison D. A. (1995). Construction and evaluation of new drug-resistance cassettes for gene disruption mutagenesis in *Streptococcus pneumoniae*, using an ami test platform. Gene 164, 123–128. 10.1016/0378-1119(95)00485-O 7590300

[B11] Comte S. Guibaud G. Baudu M. (2007). Effect of extraction method on EPS from activated sludge: an HPSEC investigation. J. Hazard. Mater. 140, 129–137. 10.1016/j.jhazmat.2006.06.058 16879910

[B12] Cross S. E. Kreth J. Zhu L. Sullivan R. Shi W. Qi F.. (2007). Nanomechanical properties of glucans and associated cell-surface adhesion of *Streptococcus mutans* probed by atomic force microscopy under *in situ* conditions. Microbiology 153, 3124–3132. 10.1099/mic.0.2007/007625-0 17768255

[B13] Cuzzi B. Herasimenka Y. Silipo A. Lanzetta R. Liut G. Rizzo R.. (2014). Versatility of the *Burkholderia cepacia* complex for the biosynthesis of exopolysaccharides: a comparative structural investigation. PLoS ONE 9:e94372. 10.1371/journal.pone.0094372 24722641 PMC3983119

[B14] Duque C. Stipp R. N. Wang B. Smith D. J. Höfling J. F. Kuramitsu H. K.. (2011). Downregulation of GbpB, a component of the VicRK regulon, affects biofilm formation and cell surface characteristics of *Streptococcus mutans*. Infect. Immun. 79, 786–796. 10.1128/IAI.00725-10 21078847 PMC3028841

[B15] Duus J. Gotfredsen C. H. Bock K. (2000). Carbohydrate structural determination by NMR spectroscopy: modern methods and limitations. Chem. Rev. 100, 4589–4614. 10.1021/cr990302n 11749359

[B16] Fabret C. Hoch J. A. (1998). A two-component signal transduction system essential for growth of *Bacillus subtilis*: implications for anti-infective therapy. J. Bacteriol. 180, 6375–6383 9829949 10.1128/jb.180.23.6375-6383.1998 PMC107725

[B17] Fujii M. Sato Y. Ito H. Masago Y. Omura T. (2012). Monosaccharide composition of the outer membrane lipopolysaccharide and O-chain from the freshwater cyanobacterium *Microcystis aeruginosa* NIES-87. J. Appl. Microbiol. 113, 896–903. 10.1111/j.1365-2672.2012.05405.x 22817604

[B18] Fukuchi K. Kasahara Y. Asai K. Kobayashi K. Moriya S. Ogasawara N. (2000). The essential two-component regulatory system encoded by yycF and yycG modulates expression of the ftsAZ operon in *Bacillus subtilis*. Microbiology 146, 1573–1583. 10.1099/00221287-146-7-1573 10878122

[B19] Hanada N. Kuramitsu H. K. (1988). Isolation and characterization of the *Streptococcus mutans gtfC* gene, coding for synthesis of both soluble and insoluble glucans. Infect. Immun. 56, 1999–2005. 2969375 10.1128/iai.56.8.1999-2005.1988 PMC259514

[B20] Hayacibara M. F. Koo H. Vacca-Smith A. M. Kopec L. K. Scott-Anne K. Cury J. A.. (2004). The influence of mutanase and dextranase on the production and structure of glucans synthesized by streptococcal glucosyltransferases. Carbohydr. Res. 339, 2127–2137. 10.1016/j.carres.2004.05.031 15280057

[B21] Iijima M. Muguruma T. Brantley W. Choe H. C. Nakagaki S. Alapati S. B.. (2012). Effect of coating on properties of esthetic orthodontic nickel-titanium wires. Angle Orthod. 82, 319–325. 10.2319/021511-112.1 21827235 PMC8867949

[B22] Ivanov I. E. Kintz E. N. Porter L. A. Goldberg J. B. Burnham N. A. Camesano T. A. (2011). Relating the physical properties of *Pseudomonas aeruginosa* lipopolysaccharides to virulence by atomic force microscopy. J. Bacteriol. 193, 1259–1266. 10.1128/JB.01308-10 21148734 PMC3067594

[B23] Kazor C. E. Mitchell P. M. Lee A. M. Stokes L. N. Loesche W. J. Dewhirst F. E.. (2003). Diversity of bacterial populations on the tongue dorsa of patients with halitosis and healthy patients. J. Clin. Microbiol. 41, 558–563. 10.1128/JCM.41.2.558-563.2003 12574246 PMC149706

[B24] Keyes P. H. (1958). Dental caries in the molar teeth of rats. I. Distribution of lesions induced by high-carbohydrate low-fat diets. J. Dent. Res. 37, 1077–1087. 10.1177/00220345580370060801 13611122

[B25] Khalikova E. Susi P. Usanov N. Korpela T. (2003). Purification and properties of extracellular dextranase from a Bacillus sp. J. Chromatogr. B Analyt. Technol. Biomed. Life Sci. 796, 315–326. 10.1016/j.jchromb.2003.08.037 14581071

[B26] Klein M. I. DeBaz L. Agidi S. Lee H. Xie G. Lin A. H.. (2010). Dynamics of *Streptococcus mutans* transcriptome in response to starch and sucrose during biofilm development. PLoS ONE 5:e13478. 10.1371/journal.pone.0013478 20976057 PMC2957427

[B27] Koo H. Hayacibara M. F. Schobel B. D. Cury J. A. Rosalen P. L. Park Y. K.. (2003). Inhibition of *Streptococcus mutans* biofilm accumulation and polysaccharide production by apigenin and tt-farnesol. J. Antimicrob. Chemother. 52, 782–789. 10.1093/jac/dkg449 14563892

[B28] Koo H. Rosalen P. L. Cury J. A. Park Y. K. Ikegaki M. Sattler A. (1999). Effect of *Apis mellifera* propolis from two Brazilian regions on caries development in desalivated rats. Caries Res. 33, 393–400. 10.1159/000016539 10460964

[B29] Kroes I. Lepp P. W. Relman D. A. (1999). Bacterial diversity within the human subgingival crevice. Proc. Natl. Acad. Sci. U.S.A. 96, 14547–14552. 10.1073/pnas.96.25.14547 10588742 PMC24473

[B30] Kruppa M. D. Lowman D. W. Chen Y. H. Selander C. Scheynius A. Monteiro M. A.. (2009). Identification of (1 → 6)-β-d-glucan as the major carbohydrate component of the *Malassezia sympodialis* cellwall. Carbohydr. Res. 344, 2474–2479. 10.1016/j.carres.2009.09.029 19853245 PMC2783858

[B31] Lathe G. H. Ruthven C. R. J. (1956). The separation of substance and estimation of their relative molecular sizes by the use of columns of starch in water. Biochem. J. 62, 665–674. 10.1042/bj0620665 13315231 PMC1215979

[B32] Lau P. C. Sung C. K. Lee J. H. Morrison D. A. Cvitkovitch D. G. (2002). PCR ligation mutagenesis in transformable streptococci: application and efficiency. J. Microbiol. Methods 49, 193–205. 10.1016/S0167-7012(01)00369-4 11830305

[B33] Lemos J. A. Burne R. A. (2008). A model of efficiency: stress tolerance by *Streptococcus mutans*. Microbiology 154(Pt 11), 3247–3255. 10.1099/mic.0.2008/023770-0 18957579 PMC2627771

[B34] Loesche W. J. (1986). Role of *Streptococcus mutans* in human dental decay. Microbiol. Rev. 50, 353–380. 3540569 10.1128/mr.50.4.353-380.1986 PMC373078

[B35] Lynch D. J. Fountain T. L. Mazurkiewicz J. E. Banas J. A. (2007). Glucan-binding proteins are essential for shaping *Streptococcus mutans* biofilm architecture. FEMS Microbiol. Lett. 268, 158–165. 10.1111/j.1574-6968.2006.00576.x 17214736 PMC1804096

[B36] Maehara H. Iwami Y. Mayanagi H. Takahashi N. (2005). Synergistic inhibition by combination of fluoride and xylitol on glycolysis by mutans streptococci and its biochemical mechanism. Caries Res. 39, 521–528. 10.1159/000088190 16251799

[B37] Mao W. Li H. Li Y. Zhang H. Qi X. Sun H.. (2009). Chemical characteristic and anticoagulant activity of the sulfated polysaccharide isolated from *Monostroma latissimum* (Chlorophyta). Int. J. Biol. Macromol. 44, 70–74. 10.1016/j.ijbiomac.2008.10.003 19007806

[B38] Mei L. Ren Y. Busscher H. J. Chen Y. van der Mei H. C. (2009). Poisson analysis of streptococcal bond-strengthening on saliva-coated enamel. J. Dent. Res. 88, 841–845. 10.1177/0022034509342523 19767582

[B39] Mei Y. Zhu H. Hu Q. Liu Y. Zhao S. Peng N.. (2015). A novel polysaccharide from mycelia of cultured *Phellinus linteus* displays antitumor activity through apoptosis. Carbohydr. Polym. 124, 90–97. 10.1016/j.carbpol.2015.02.009 25839798

[B40] Morrison D. A. Cvitkovitch D. G. (2002). PCR ligation mutagenesis in transformable streptococci: application and efficiency. J. Microbiol. Methods 49, 193–205. 10.1016/S0167-7012(01)00369-4 11830305

[B41] Ng W. L. Robertson G. T. Kazmierczak K. M. Zhao J. Gilmour R. Winkler M. E. (2003). Constitutive expression of PcsB suppresses the requirement for the essential VicR (YycF) response regulator in *Streptococcus pneumoniae* R6. Mol. Microbiol. 50, 1647–1663. 10.1046/j.1365-2958.2003.03806.x 14651645

[B42] Raghavan V. Groisman E. A. (2010). Orphan and hybrid two-component system proteins in health and disease. Curr. Opin. Microbiol. 13, 226–231. 10.1016/j.mib.2009.12.010 20089442 PMC2861427

[B43] Rolla G. Ciardi J. E. Schultz S. A. (1983). Adsorption of glucosyltransferase to saliva coated hydroxyapatite. Possible mechanism for sucrose dependent bacterial colonization of teeth. Scand. J. Dent. Res. 91, 112–117. 10.1111/j.1600-0722.1983.tb00786.x 6304864

[B44] Rozen R. Steinberg D. Bachrach G. (2004). *Streptococcus mutans* fructosyltransferase interactions with glucans. FEMS Microbiol. Lett. 232, 39–43. 10.1016/S0378-1097(04)00065-5 15019732

[B45] Senadheera D. Krastel K. Mair R. Persadmehr A. Abranches J. Burne R. A.. (2009). Inactivation of VicK affects acid production and acid survival of *Streptococcus* mutans. J. Bacteriol. 191, 6415–6424. 10.1128/JB.00793-09 19684142 PMC2753040

[B46] Senadheera M. D. Guggenheim B. Spatafora G. A. Huang Y. C. Choi J. Hung D. C.. (2005). A VicRK signal transduction system in *Streptococcus mutans* affects *gtfBCD, gbpB*, and *ftf* expression, biofilm formation, and genetic competence development. J. Bacteriol. 187, 4064–4076. 10.1128/JB.187.12.4064-4076.2005 15937169 PMC1151735

[B47] Senadheera M. D. Lee A. W. Hung D. C. Spatafora G. A. Goodman S. D. Cvitkovitch D. G. (2007). The *Streptococcus mutans vicX* gene product modulates *gtfB/C* expression, biofilm formation, genetic competence, and oxidative stress tolerance. J. Bacteriol. 189, 1451–1458. 10.1128/JB.01161-06 17114248 PMC1797355

[B48] Seo M. J. Kang B. W. Park J. U. Kim M. J. Lee H. H. Choi Y. H.. (2011). Biochemical characterization of the exopolysaccharide purified from *Laetiporus sulphureus* mycelia. J. Microbiol. Biotechnol. 21, 1287–1293. 10.4014/jmb.1106.06046 22210615

[B49] Shemesh M. Tam A. Steinberg D. (2007). Expression of biofilm-associated genes of *Streptococcus mutans* in response to glucose and sucrose. J. Med. Microbiol. 56, 1528–1535. 10.1099/jmm.0.47146-0 17965356

[B50] Stewart P. S. Franklin M. J. (2008). Physiological heterogeneity in biofilms. Nat. Rev. Microbiol. 6, 199–210. 10.1038/nrmicro1838 18264116

[B51] Stipp R. N. Boisvert H. Smith D. J. Höfling J. F. Duncan M. J. Mattos-Graner R. O. (2013). CovR and VicRK regulate cell surface biogenesis genes required for biofilm formation in *Streptococcus mutans*. PLoS ONE 8:e58271. 10.1371/journal.pone.0058271 23554881 PMC3595261

[B52] Su L. K. Yu F. Li Z. F. Zeng C. Xu Q. A. Fan M. W. (2014). Intranasal co-delivery of IL-6 gene enhances the immunogenicity of anti-caries DNA vaccine. Acta Pharmacol. Sin. 35, 592–598. 10.1038/aps.2013.184 24705100 PMC4814028

[B53] Szurmant H. Mohan M. A. Imus P. M. Hoch J. A. (2007). YycH and YycI interact to regulate the essential YycFG two-component system in *Bacillus subtilis*. J. Bacteriol. 189, 3280–3289. 10.1128/JB.01936-06 17307850 PMC1855854

[B54] Thurnheer T. van der Ploeg J. R. Giertsen E. Guggenheim B. (2006). Effects of *Streptococcus mutans gtfC* deficiency on mixed oral biofilms *in vitro*. Caries Res. 40, 163–171. 10.1159/000091065 16508276

[B55] Toukach F. V. Ananikov V. P. (2013). Recent advances in computational predictions of NMR parameters for the structure elucidation of carbohydrates: methods and limitations. Chem. Soc. Rev. 42, 8376–8415. 10.1039/c3cs60073d 23887200

[B56] Wagner C. Saizieu Ad. Ad. Schönfeld H. J. Kamber M. Lange R. Thompson C. J.. (2002). Genetic analysis and functional characterization of the *Streptococcus pneumoniae* vic operon. Infect. Immun. 70, 6121–6128. 10.1128/IAI.70.11.6121-6128.2002 12379689 PMC130280

[B57] Xiao J. Klein M. I. Falsetta M. L. Lu B. Delahunty C. M. Yates J. R. III. (2012). The exopolysaccharide matrix modulates the interaction between 3D architecture and virulence of a mixed-species oral biofilm. PLoS Pathog. 8:e1002623. 10.1371/journal.ppat.1002623 22496649 PMC3320608

[B58] Xiao J. Koo H. (2010). Structural organization and dynamics of exopolysaccharide matrix and microcolonies formation by *Streptococcus mutans* in biofilms. J. Appl. Microbiol. 108, 2103–2113. 10.1111/j.1365-2672.2009.04616.x 19941630

[B59] Xu X. Zhou X. D. Wu C. D. (2012). Tea catechin epigallocatechin gallate inhibits *Streptococcus mutans* biofilm formation by suppressing *gtf* genes. Arch. Oral Biol. 57, 678–683. 10.1016/j.archoralbio.2011.10.021 22169220

[B60] Yamashita Y. Bowen W. H. Burne R. A. Kuramitsu H. K. (1993). Role of the *Streptococcus mutans gtf* genes in caries induction in the specific-pathogen-free rat model. Infect. Immun. 61, 3811–3817. 8359902 10.1128/iai.61.9.3811-3817.1993 PMC281081

[B61] Yang Y. M. Jiang D. Qiu Y. X. Fan R. Zhang R. Ning M. Z.. (2013). Effects of combined exogenous dextranase and sodium fluoride on *Streptococcus mutans* 25175 monospecies biofilms. Am. J. Dent. 26, 239–243. 24479273

[B62] Zheng X. Zhang K. Zhou X. Liu C. Li M. Li Y.. (2013). Involvement of gshAB in the interspecies competition within oral biofilm. J. Dent. Res. 92, 819–824. 10.1177/0022034513498598 23872989

