# Supplementary figures and images for: Modulation of Biofilm Exopolysaccharides by the Streptococcus mutans vicX Gene
Source: Front Microbiol. 2015 Dec 21;6:1432. doi: 10.3389/fmicb.2015.01432 (PMC4685068; doi:10.3389/fmicb.2015.01432)

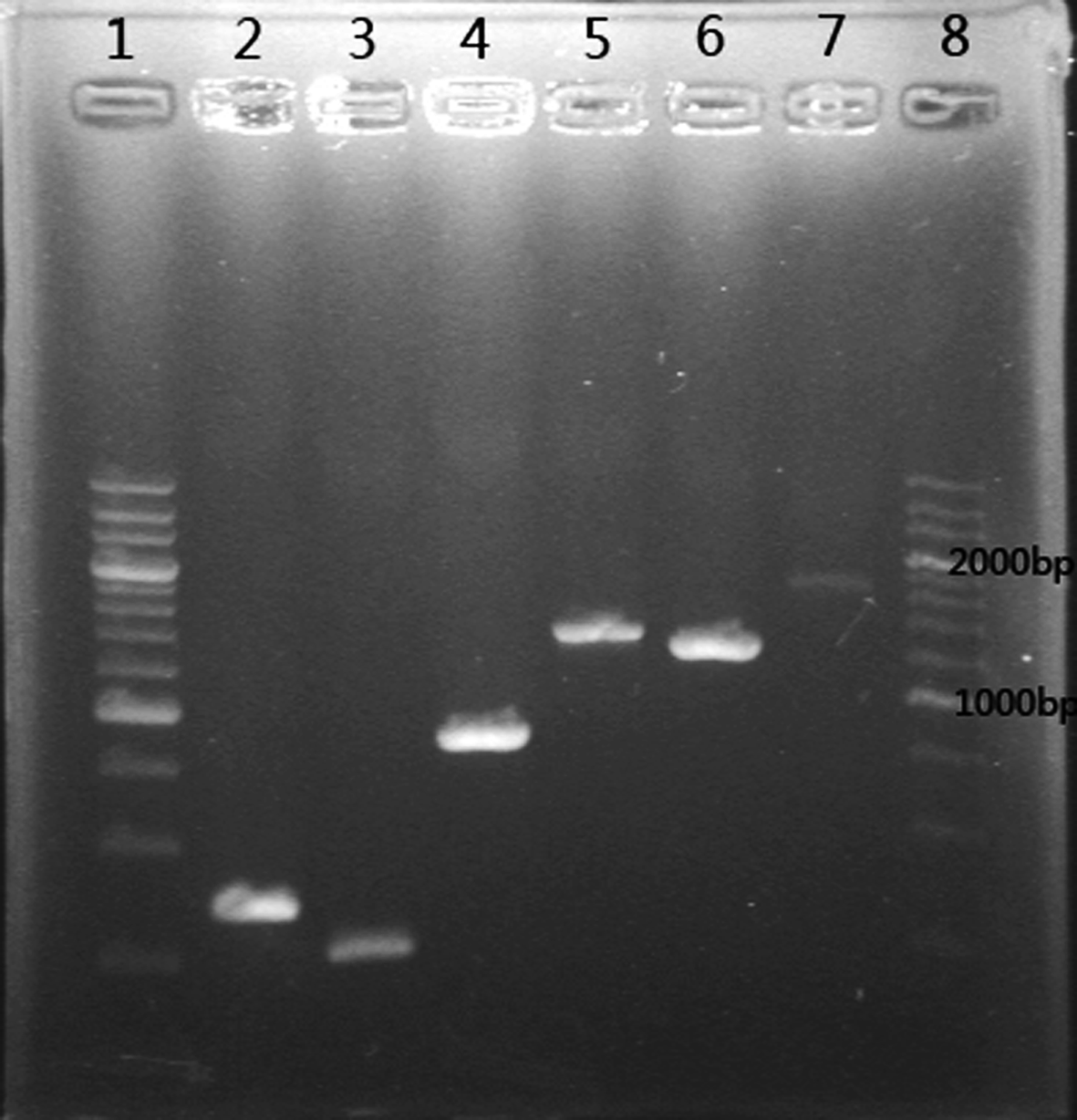

Supplement: Figure S1 — PCR fragment analysis in the vicX mutant (1) DNA marker; (2) P1–P2 PCR fragment, WT template (478 bp); (3) P3–P4 PCR fragment, WT template (401 bp); (4) erythromycin (erm) cassette PCR confirmation fragment, mutant template (876 bp); (5) P1–PR PCR confirmation fragment, M template (1354 bp); (6) PF–P4 PCR confirmation fragment, mutant template (1279 bp); (7) P1–P4 PCR fragment for confirmation, mutant template (1760 bp); (8) DNA marker. [file Image1.TIF]

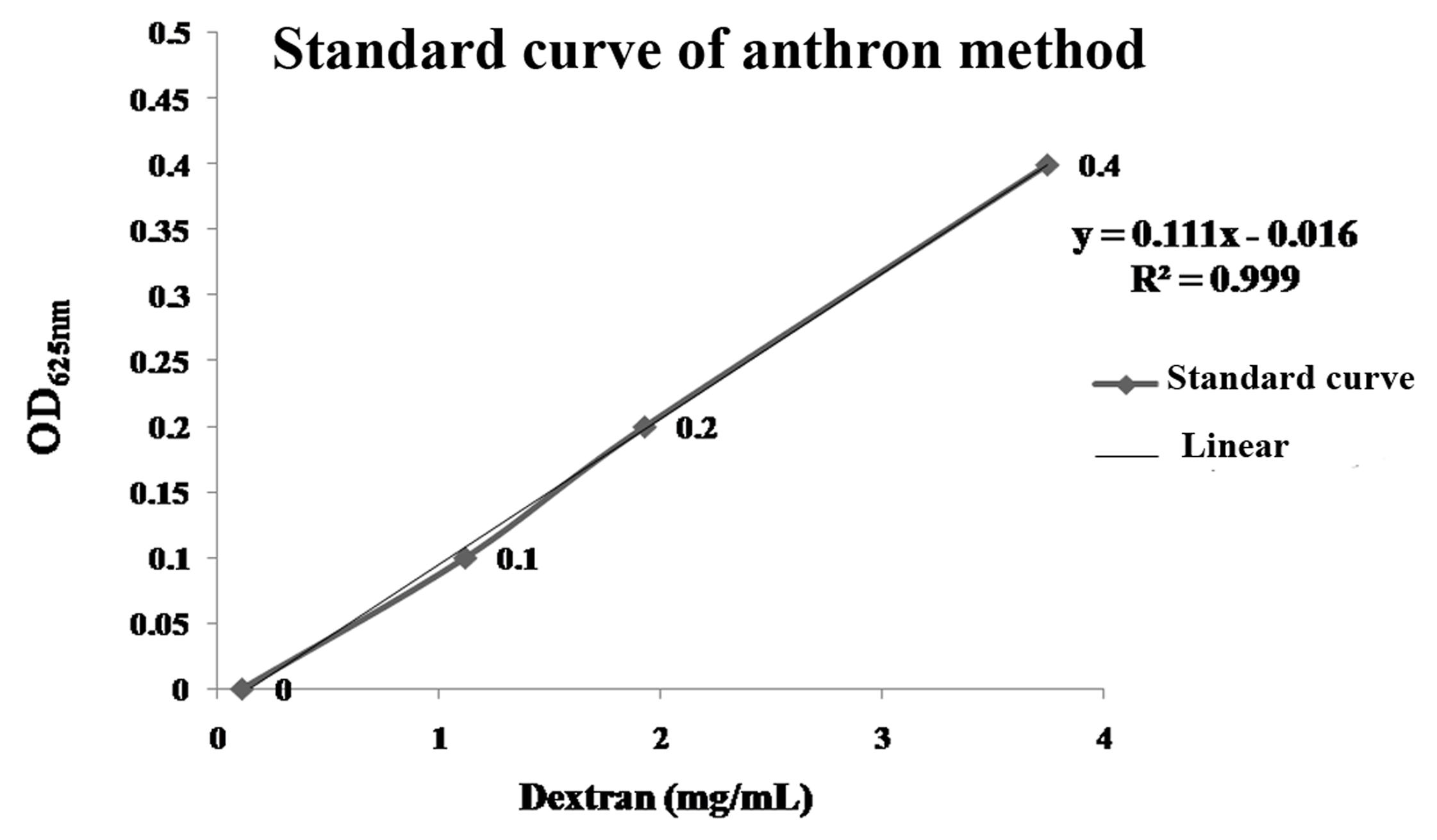

Supplement: Figure S2 — Standard curve for the anthrone method, generated based on known concentrations of dextran. [file Image2.TIF]

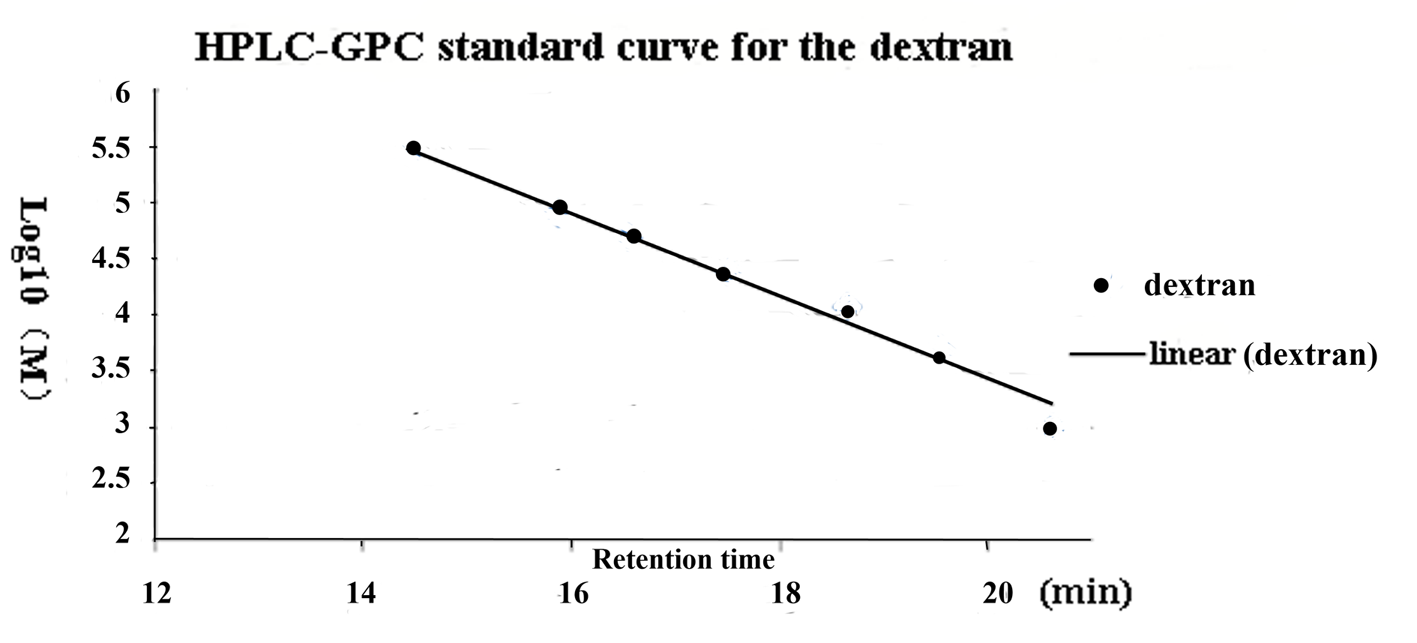

Supplement: Figure S3 — Standard curve of high-performance gel permeation chromatography (GPC), for determination of the molecular weight of the dextran; the linear correlation between the logMw and retention time was calculated (y = −0.369x + 10.80; R2 = 0.978). [file Image3.TIF]

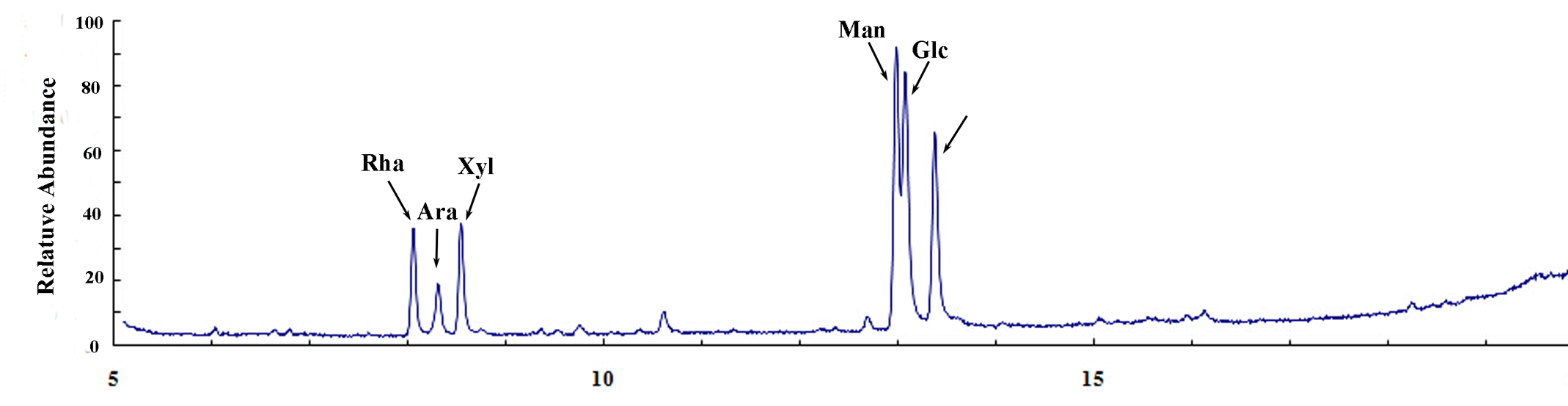

Supplement: Figure S4 — GC/MS chromatogram of six standard monosaccharides for monosaccharide composition analysis of WIG: (1) rhamnose (Rha, retention time: 8.030 min); (2) arabinose (Ara, retention time: 8.270 min); (3) Xylose (Xyl, retention time: 8.514 min); (4) mannose (Man, retention time: 12.956 min); (5) glucose (Glc, retention time: 13.066 min); and (6) galactose (Gal, retention time: 13.346 min). [file Image4.TIF]

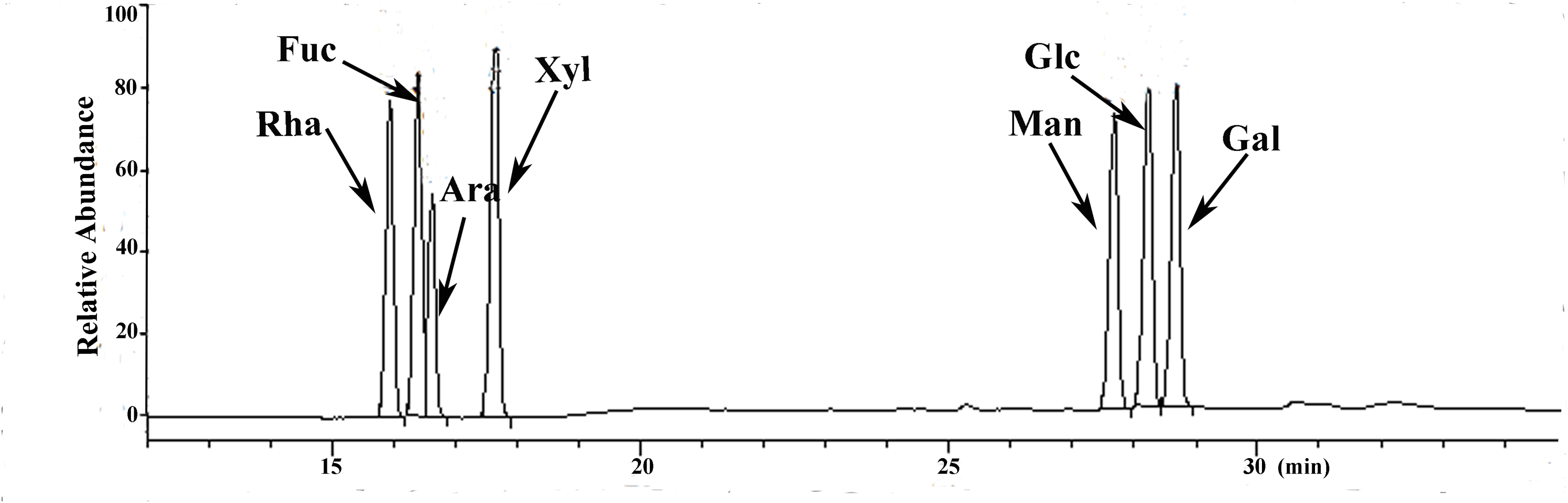

Supplement: Figure S5 — GC/MS chromatogram of seven standard monosaccharides for monosaccharide composition analysis of WSG: (1) rhamnose (Rha, retention time: 15.951 min); (2) fructose (Fuc, retention time: 16.408 min); (3) arabinose (Ara, retention time: 16.631 min); (4) Xylose (Xyl, retention time: 17.860 min); (5) mannose (Man, retention time: 27.690 min); (6) glucose (Glc, retention time: 28.243min); and (7) galactose (Gal, retention time: 28.694 min). [file Image5.TIF]

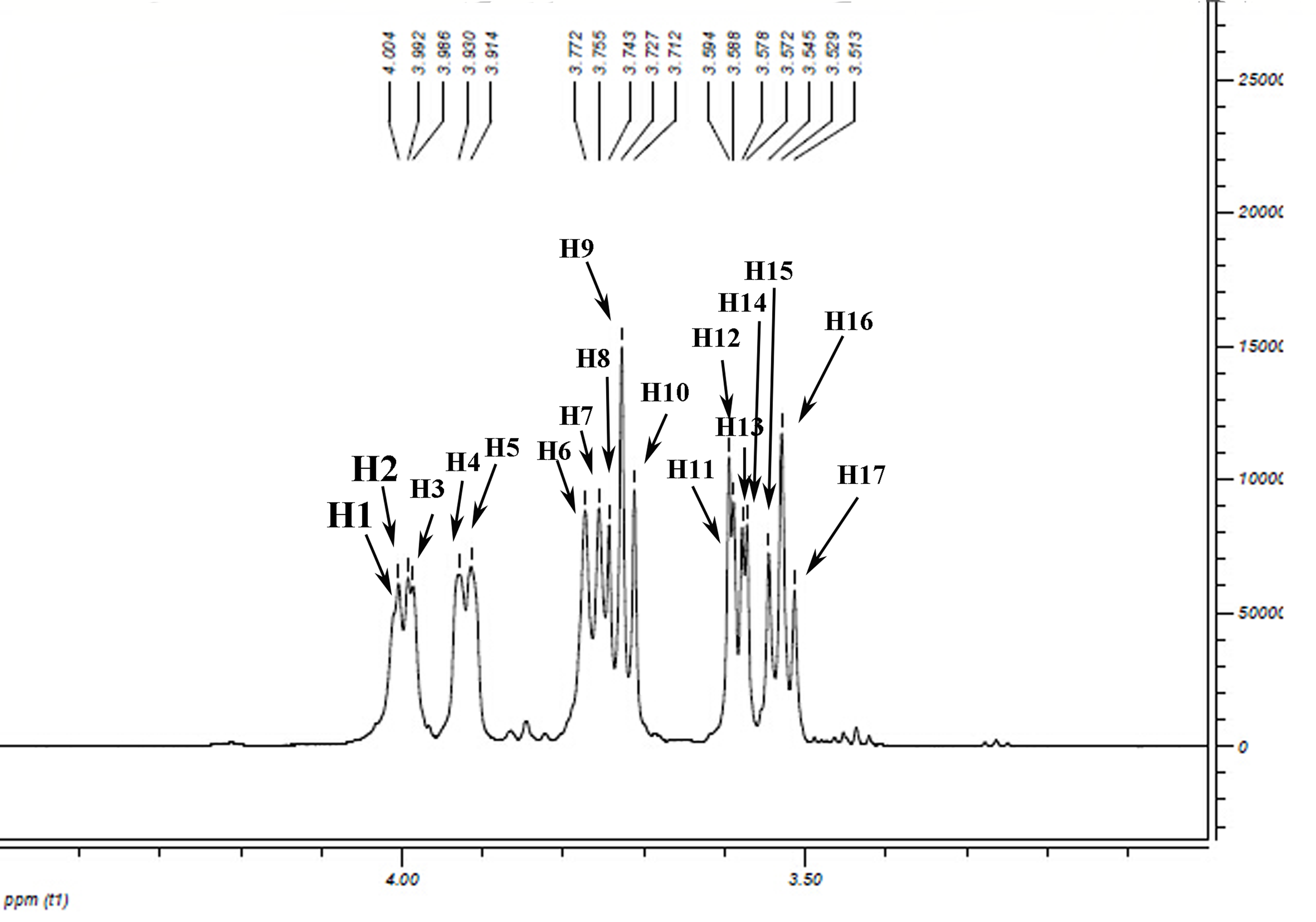

Supplement: Figure S6 — Several sharp and well-resolved peaks corresponding to the dextran standard metabolites could be observed at 600 MHz 1H nuclear magnetic resonance (NMR) spectroscopy (0.01% Deuterium generation of trimethyl silane sodium propionate as an internal control). The 1D 1H NMR spectrum for each of the strains consisted mostly of signals at 4.003 (H1), 3.992 (H2), 3.985 (H3), 3.9301 (H4), 3.913 (H5), 3.772 (H6), 3.755 (H7), 3.743 (H8), 3.727 (H9), 3.711 (H10), 3.594 (H11), 3.588 (H12), 3.577 (H13), 3.571 (H14), 3.544 (H15), 3.528 (H16), and 3.513 (H17) ppm. [file Image6.TIF]

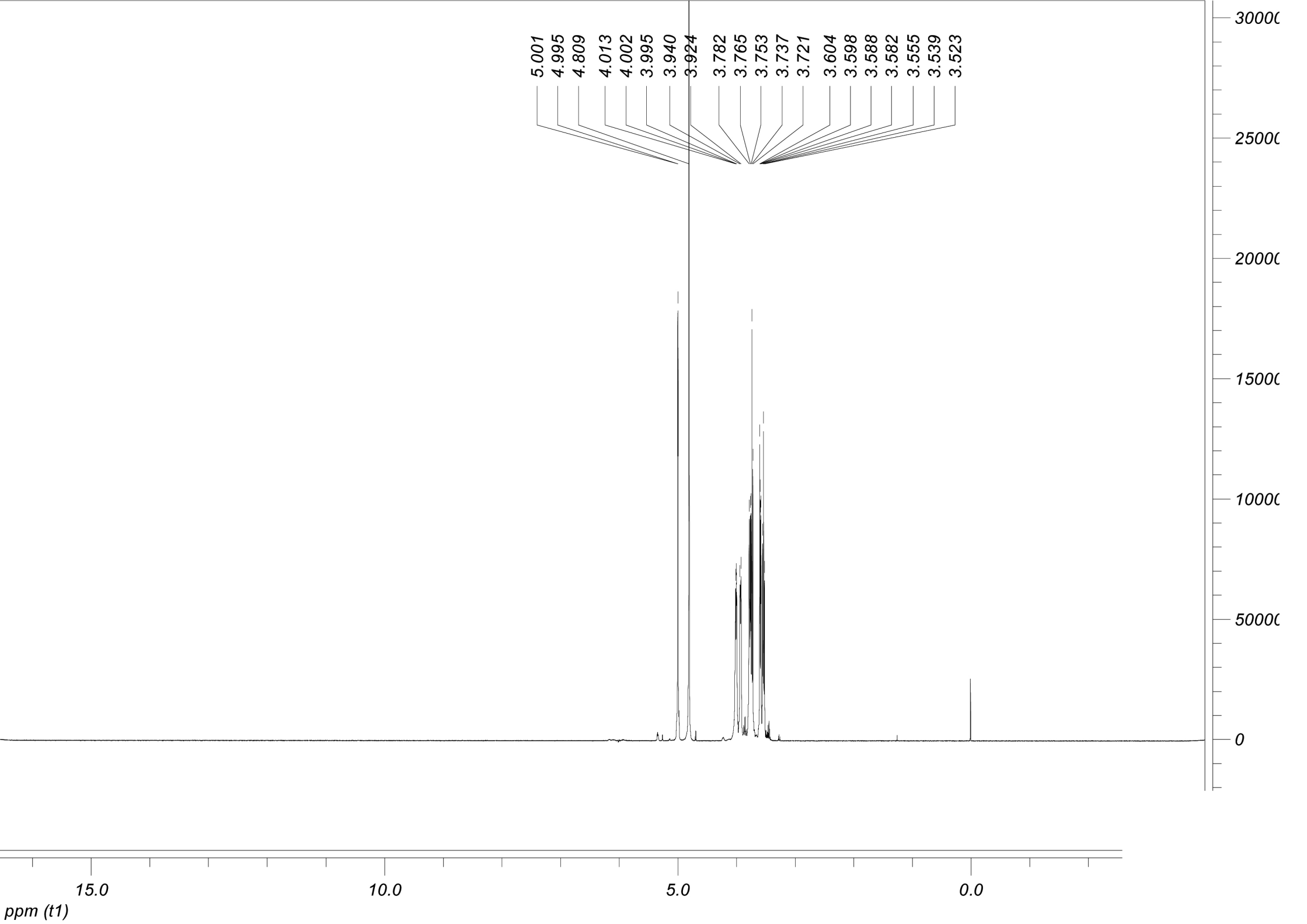

Supplement: Supplementary file 8 [file DataSheet2.ZIP › 1H-NMR data/Glucan 1H NMR/Glucan 1H-NMR.pdf]

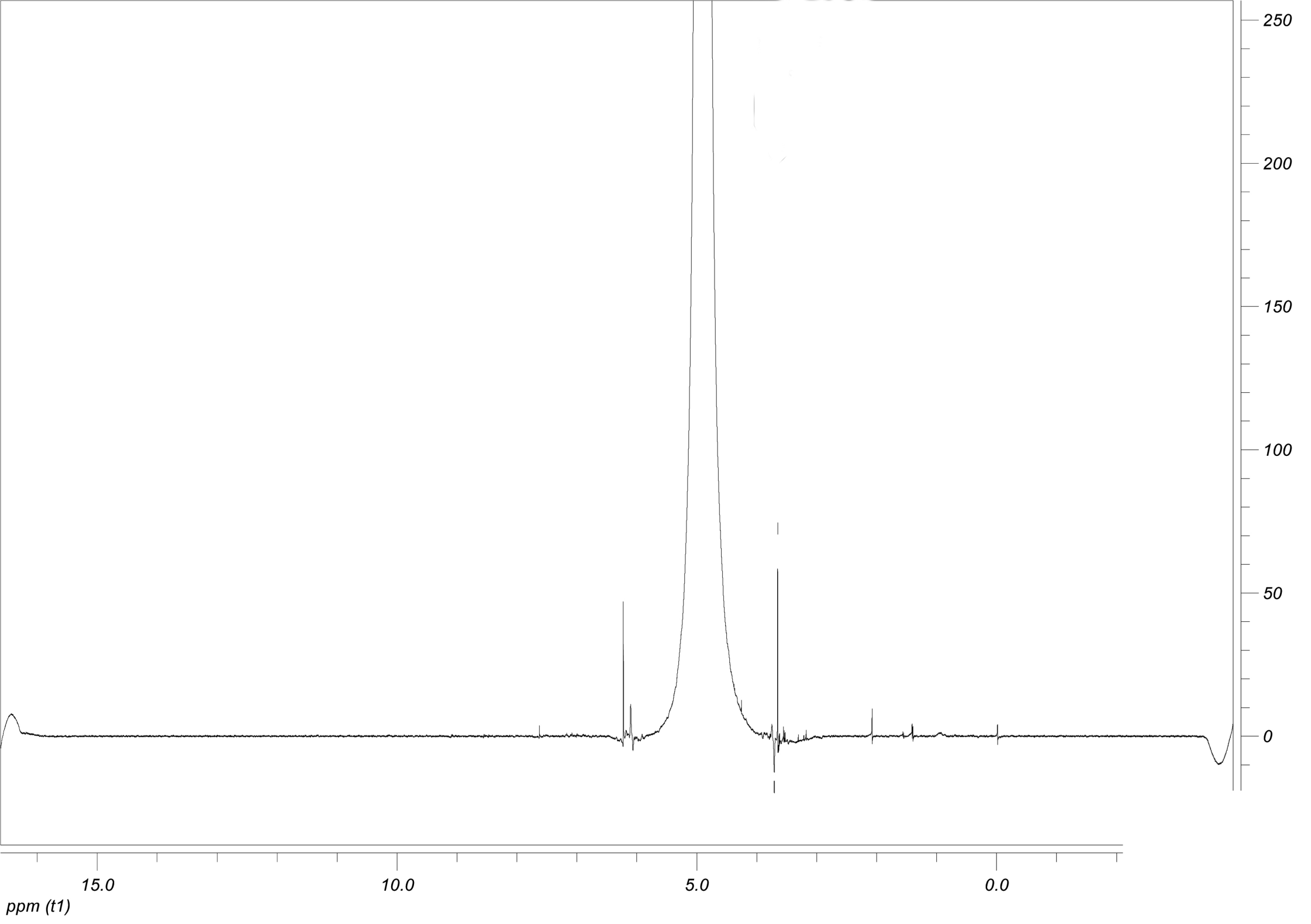

Supplement: Supplementary file 8 [file DataSheet2.ZIP › 1H-NMR data/SmuvicX WIG 1H NMR/SmuvicX WIG 1H NMR.pdf]

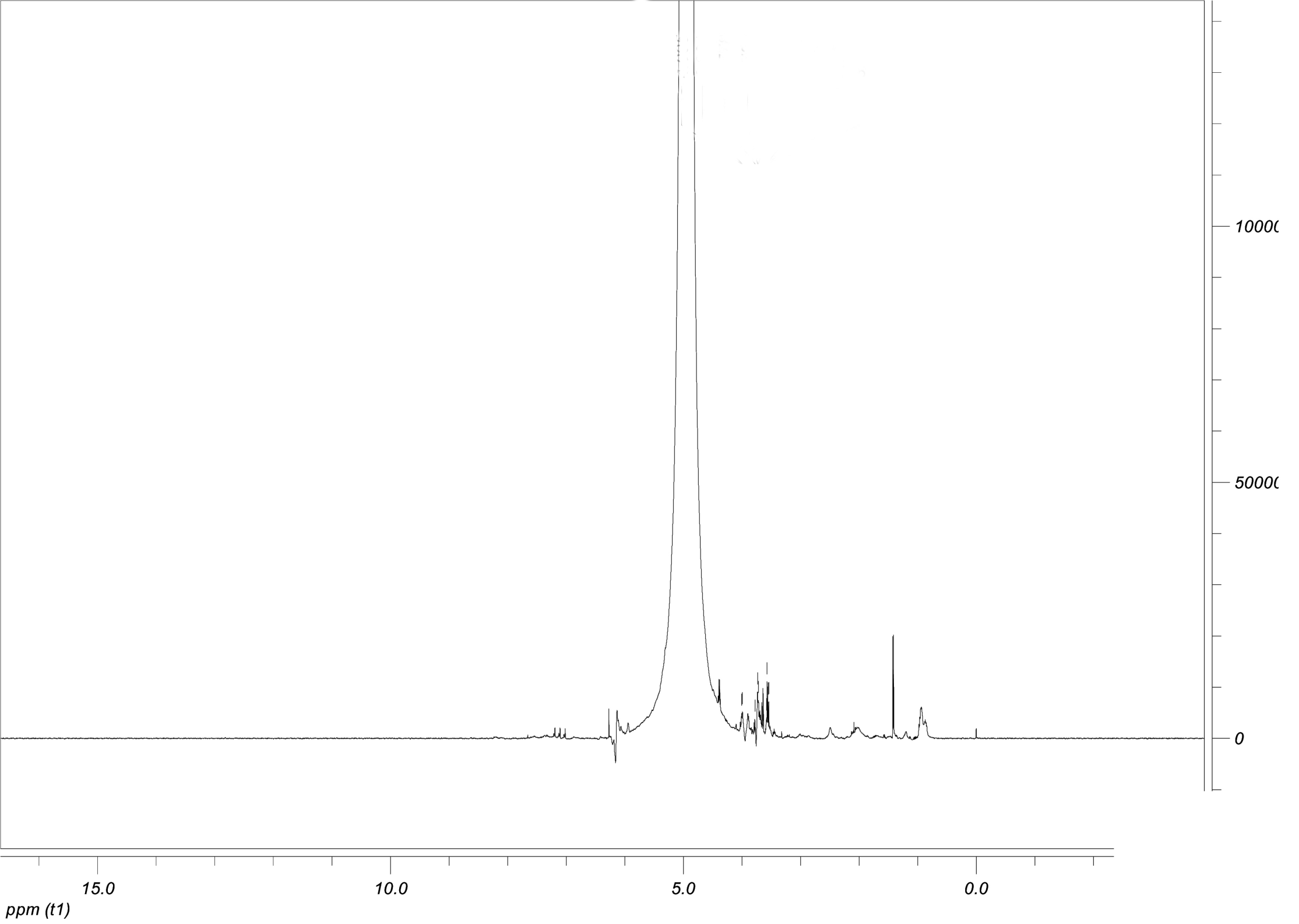

Supplement: Supplementary file 8 [file DataSheet2.ZIP › 1H-NMR data/SmuvicX WSG 1HNMR/SmuvicX WSG 1HNMR.pdf]

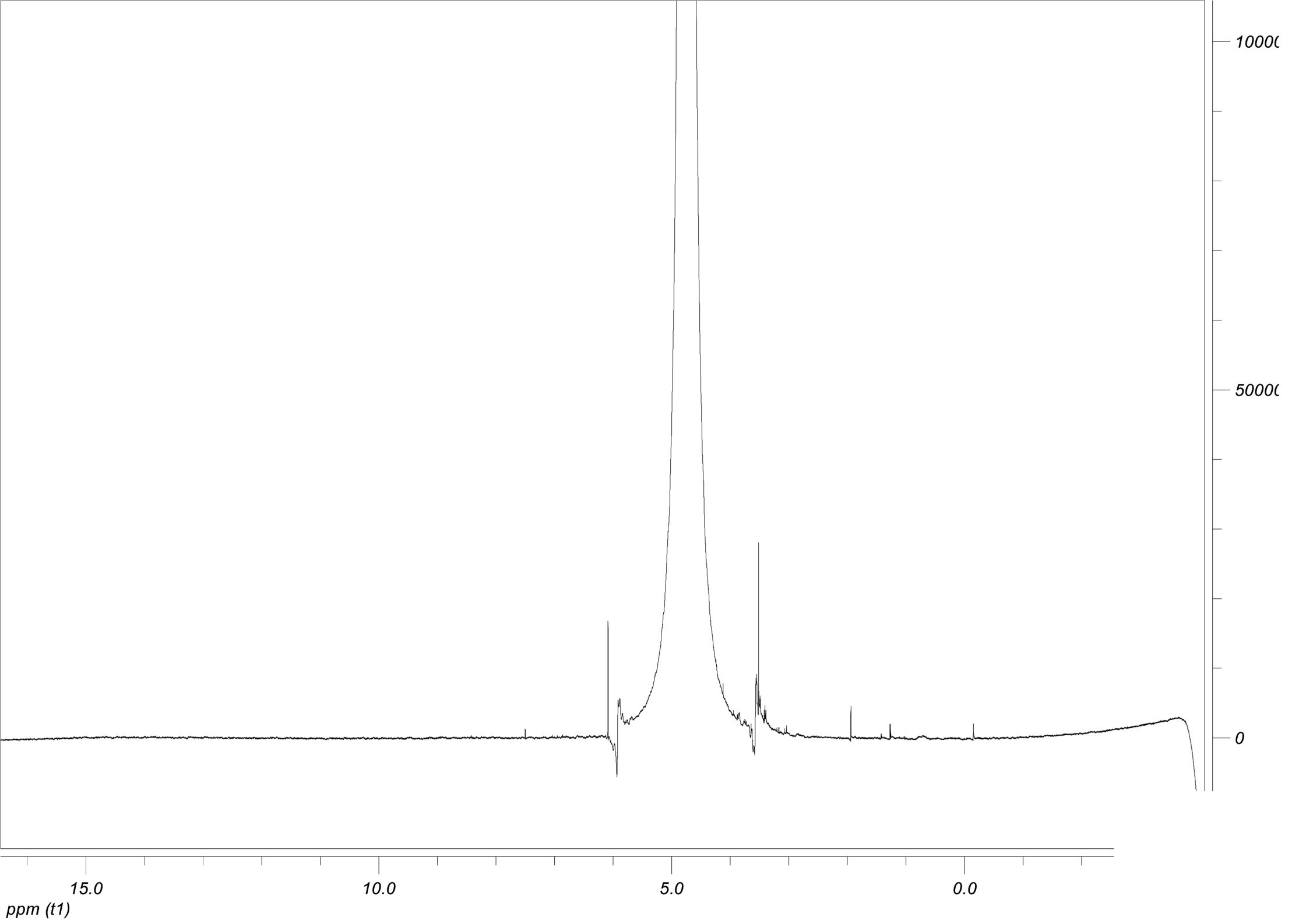

Supplement: Supplementary file 8 [file DataSheet2.ZIP › 1H-NMR data/SmuvicX+ WIG 1H NMR/SmuvicX+ WIG 1HNMR.pdf]

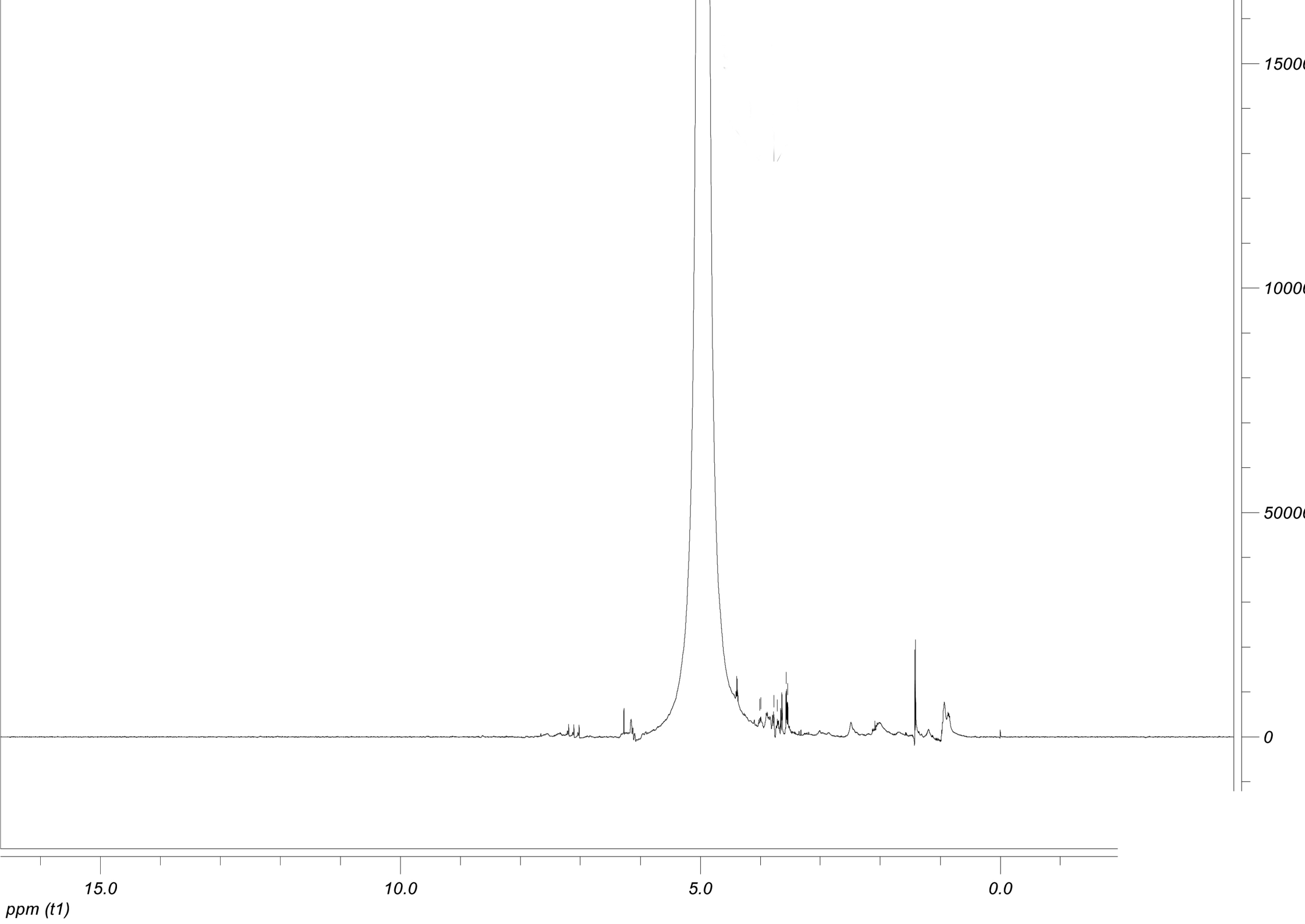

Supplement: Supplementary file 8 [file DataSheet2.ZIP › 1H-NMR data/SmuvicX+ WSG 1H NMR/SmuvicX+WSG 1H NMR.pdf]

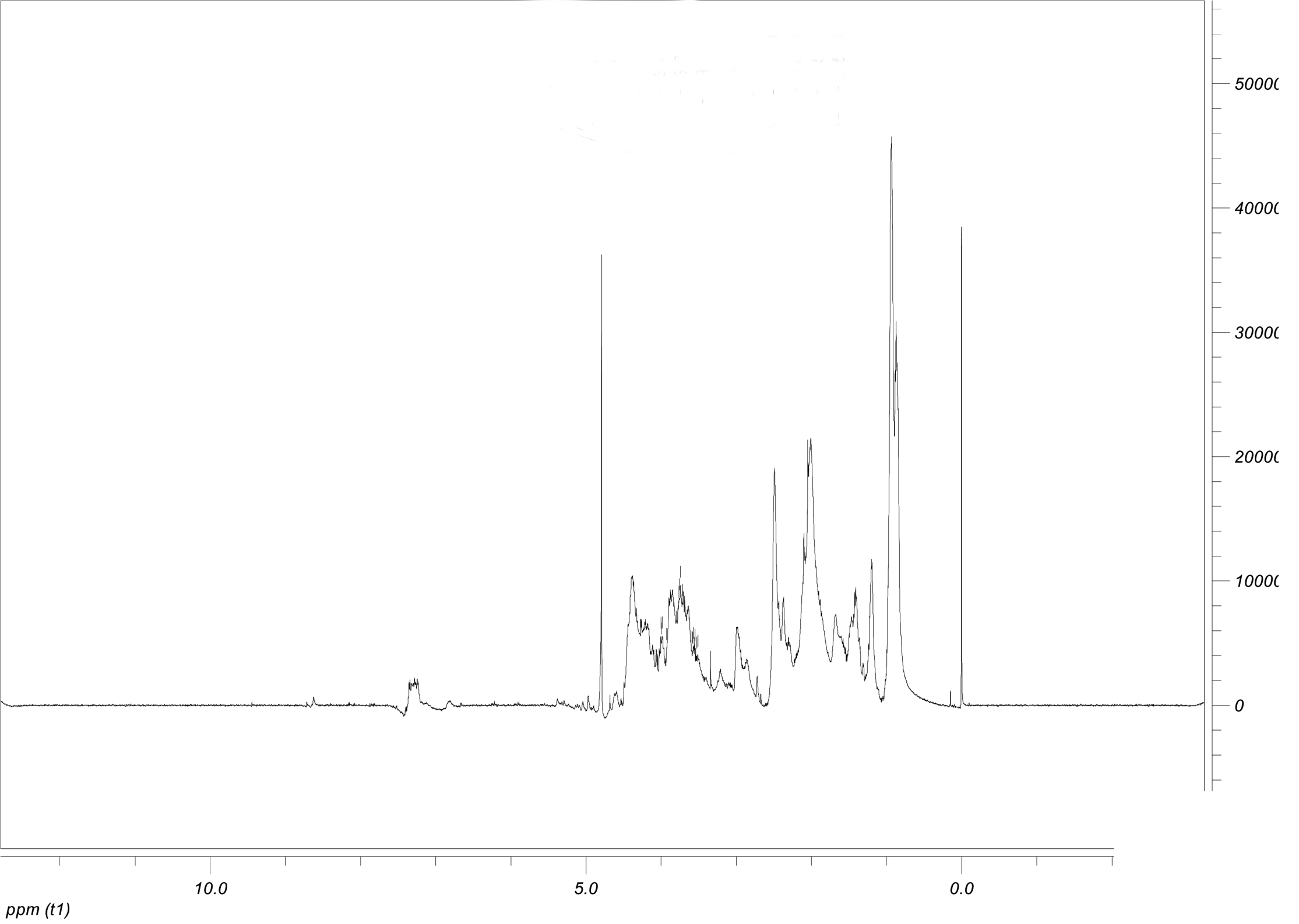

Supplement: Supplementary file 8 [file DataSheet2.ZIP › 1H-NMR data/UA159WIG 1H NMR/UA159WIG1HNMR.pdf]

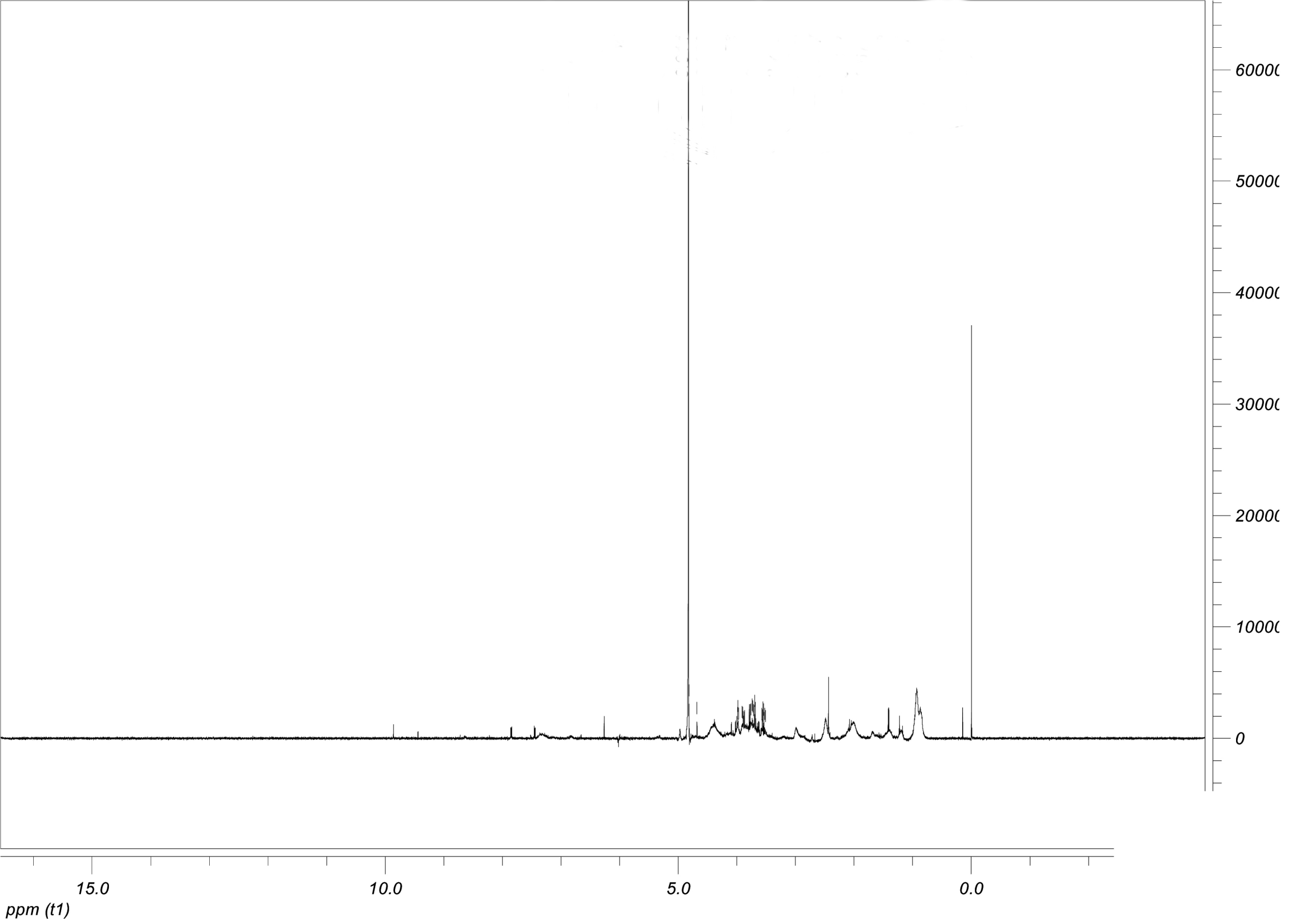

Supplement: Supplementary file 8 [file DataSheet2.ZIP › 1H-NMR data/UA159WSG 1HNMR/UA159WSG 1H NMR.pdf]
