# Supplementary material for: Modulation of Biofilm Exopolysaccharides by the Streptococcus mutans vicX Gene
Source: Front Microbiol. 2015 Dec 21;6:1432. doi: 10.3389/fmicb.2015.01432 (PMC4685068; doi:10.3389/fmicb.2015.01432)
Supplement: Supplementary file 7 [file DataSheet1.ZIP › SmuvicX_DNA_sequencing/SmuvicX_DNA_sequencing_file1.pdf]

CDS9241_CDS8908U 1 TGATGTCTAG GAAAAAGAAG GTTTATATGA AAACATTAGA AAAAAAACTG

CDS9241_CDS8908U 51 GCAGAAGACT TTAAGATCGT CTTTTCTGAC AAGGAATTAT TGGAAACTGC

CDS9241_CDS8908U 101 CTTTACTCAT ACTAGTTATG CTAATGAGCA TCGCCTCCTA AACATTTCAC

CDS9241_CDS8908U 151 ATAACGAGCG CTTGGAATTT TTAGGAGACG CTGTTCTGCA GTTAACGATT

CDS9241_CDS8908U 201 TCACATTATC TTTTTGACAA ATACCCTCAA AAAGCTGAAG GTGATTTATC

CDS9241_CDS8908U 251 AAAAATGCGT TCGATGATTG TTCGTGAAGA AAGTTTGGCG GGTTTTTCTA

CDS9241_CDS8908U 301 GAAATTGTCA CTTTGACCGC TATATTAAAT TAGGTAAGGG TGAAGAAAAA

CDS9241_CDS8908U 351 TCTGGA

334位点

1位点
