# Supplementary material for: Modulation of Biofilm Exopolysaccharides by the Streptococcus mutans vicX Gene
Source: Front Microbiol. 2015 Dec 21;6:1432. doi: 10.3389/fmicb.2015.01432 (PMC4685068; doi:10.3389/fmicb.2015.01432)
Supplement: Supplementary file 7 [file DataSheet1.ZIP › SmuvicX_DNA_sequencing/SmuvicX_DNA_sequencing_file2.pdf]

```
CDS9241_CDS8830F 1   TCGGTATCGG TTTCTTTTAA TTCAATTGTT TTATTACTTG GTTGAGTACT
CDS9241_CDS8830F 51  TTTTCACTCG TTAAAAAGTT TTGAGAATAT TTTATATTTT TGTTCATGTA
CDS9241_CDS8830F 101 ATCACTCCTT CTTAATTACA AATTTTTAGC ATCTAATTTA ACTTCAATTC
CDS9241_CDS8830F 151 CTATTATACA AAATTTTAAG ATACAAATCA AACAAATTTT GGGCCCGGGG
CDS9241_CDS8830F 201 CGCGCCTGAT ACCTCGCCAG ATACTGCTTT ACCATTAGCA AAAATCTAGT
CDS9241_CDS8830F 251 GGACAGCTTA AAAGGAAGAA TGAAAAACAA AGATTACTCG GAAATGGTTG
CDS9241_CDS8830F 301 TTCTGGGTGA TTTTTGCTAT AATAGAAAGG TCTAAGGATA AAAGAAAGGA
CDS9241_CDS8830F 351 TTTATATGAA AACATTAGAA AAAAAACTGG CAGAAGACTT TAAGATCGTC
CDS9241_CDS8830F 401 TTTTCTGACA AGGAATTATT GGAAACTGCC TTTACTCATA CTAGTTATGC
CDS9241_CDS8830F 451 TAATGAGCAT CGCCTCCTAA ACATTTCACA TAACGAGCGC TTGGAATTTT
CDS9241_CDS8830F 501 TAGGAGACGC TGTTCTGCAG TTAACGATTT CACATTATCT TTTTGACAAA
CDS9241_CDS8830F 551 TACCCTCAAA AAGCTGAAGG TGATTTATCA AAAATGCGTT CGATGATTGT
CDS9241_CDS8830F 601 TCGTGAAGAA AGTTTGGCGG GTTTTTCTAG AAATTGTCAC TTTGACCGCT
CDS9241_CDS8830F 651 ATATTAAATT AGGTAAGGGT GAAAAAAAAA TCTGGA
```
