# Supplementary material for: Modulation of Biofilm Exopolysaccharides by the Streptococcus mutans vicX Gene
Source: Front Microbiol. 2015 Dec 21;6:1432. doi: 10.3389/fmicb.2015.01432 (PMC4685068; doi:10.3389/fmicb.2015.01432)
Supplement: Supplementary file 7 [file DataSheet1.ZIP › SmuvicX_DNA_sequencing/SmuvicX_DNA_sequencing_file3.pdf]

```
CDS9241_CDS8908U1 1    ATTTCTTCCG AaCAATCATC GAACGCATTT TTGATAAATC ACCTTCAGCT
CDS9241_CDS8908U1 51   TTTTGAGGGT ATTTGTCAAA AAGATAATGT GAAATCGTTA ACTGCAGAAC
CDS9241_CDS8908U1 101  AGCGTCTCCT AAAAATTCCA AGCGCTCGTT ATGTGAAATG TTTAGGAGGC
CDS9241_CDS8908U1 151  GATGCTCATT AGCATAACTA GTATGAGTAA AGGCAGTTTC CAATAATTCC
CDS9241_CDS8908U1 201  TTGTCAGAAA AGACGATCTT AAAGTCTTCT GCCAGTTTTT TTTCTAATGT
CDS9241_CDS8908U1 251  TTTCATATAA ATCCTTTCTT TTATCCTTAG ACCTTTCTAT TATAGCAAAA
CDS9241_CDS8908U1 301  ATCACCCAGA ACAACCATTT CCGAGTAATC TTTGTTTTTC ATTCTTCCTT
CDS9241_CDS8908U1 351  TTAAGCTGTC CACTAGATTT TTGCTAATGG TAAAGCAGTA TCTGGCGAGG
CDS9241_CDS8908U1 401  TATCAGGCGC GCCCCGGGCC CAAAATTTGT TTGATTTGTA TCTTAAAATT
CDS9241_CDS8908U1 451  TTGTATAATA GGAATTGAAG TTAAATTAGA TGCTAAAAAT TTGTAATTAA
CDS9241_CDS8908U1 501  GAAGGAGTGA TTACATGAAC AAAAATATAA AATATTCTCA AAACTTTTTA
CDS9241_CDS8908U1 551  ACGAGTGAAA AAGTACTCAA CCAAGTAATA AAACAATTGA ATTTAAAAGA
CDS9241_CDS8908U1 601  AACCGATACC GTTTACGAAA TTGGAACAGG TAAAGGGCAT TTAACGACGA
CDS9241_CDS8908U1 651  AACTGGCTAA AATAAGTAAA CAGGTAACGT CTATTGAATT AGACAGTCAT
CDS9241_CDS8908U1 701  CTATTCAACT TATCGTCAGA AAAATTAAAA CTGAATACTC GTGTCACTTT
CDS9241_CDS8908U1 751  AATTCACCAA GATATTCTAC AGTCTCAATT CCCTAACAAA CAGAGGTATA
CDS9241_CDS8908U1 801  AAATTGTTGG GAGTATTCCT TACCATTTAA GCACACAAAT TATTAAAAAA
CDS9241_CDS8908U1 851  GTGGTTTTTG AAAGCCATGC GTCTGACATC TATCTGATTG TTGAAGAAGG
CDS9241_CDS8908U1 901  ATTCTACAAG CGTACCTTGG ATATTCACCG AACACTAGGG TTGCTCTTGC
CDS9241_CDS8908U1 951  ACACTCAAGT CTCGATTCAG CATTTGCTTT AGCTGCCAGC GGATGCTTTC
CDS9241_CDS8908U1 1001 ATCCTAAACA AAAGTAAACA GTGTTCTTAT AACTTACCCG CATACACAGA
CDS9241_CDS8908U1 1051 TGTCAGATAT ATTGAAGCTA TATACGTACT TTGTTTCAAA TGGGTCAATC
CDS9241_CDS8908U1 1101 GAGAAATATC GTCACT
```
