# Supplementary material for: Modulation of Biofilm Exopolysaccharides by the Streptococcus mutans vicX Gene
Source: Front Microbiol. 2015 Dec 21;6:1432. doi: 10.3389/fmicb.2015.01432 (PMC4685068; doi:10.3389/fmicb.2015.01432)
Supplement: Supplementary file 7 [file DataSheet1.ZIP › SmuvicX_DNA_sequencing/SmuvicX_DNA_sequencing_file4.pdf]

CDS9241_CDS8830R1 1 TGACTGATTt TAGTAACAGT TGACGATATT CTCGATTGAC CCATTTTGAA

CDS9241_CDS8830R1 51 ACAAAGTACG TATATAGCTT CCAATATTTA TCTGGAACAT CTGTGGTATG

CDS9241_CDS8830R1 101 GCGGGTAAGT TTTATTAAGA CACTGTTTAC TTTTGGTTTA GGATGAAAGC

CDS9241_CDS8830R1 151 ATTCCGCTGG CAGCTTAAGC AATTGCTGAA TCGAGACTTG AGTGTGCAAG

CDS9241_CDS8830R1 201 AGCAACCCTA GTGTTCGGTG AATATCCAAG GTACGCTTGT AGAATCCTTC

CDS9241_CDS8830R1 251 TTCAACAATC AGATAGATGT CAGACGCATG GCTTTCAAAA ACCACTTTTT

CDS9241_CDS8830R1 301 TAATAATTTG TGTGCTTAAA TGGTAAGGAA TACTCCCAAC AATTTTATAC

CDS9241_CDS8830R1 351 CTCTGTTTGT TAGGGAATTG AGACTGTAGA ATATCTTGGT GAATTAAAGT

CDS9241_CDS8830R1 401 GACACGAGTA TTCAGTTTTA ATTTTTCTGA CGATAAGTTG AATAGATGAC

CDS9241_CDS8830R1 451 TGTCTAATTC AATAGACGTT ACCTGTTTAC TTATTTTAGC CAGTTTCGTC

CDS9241_CDS8830R1 501 GTTAAATGCC CTTTACCTGT TCCAATTTCG TAAACGGTAT CGGTTTCTTT

CDS9241_CDS8830R1 551 TAAATTCAAT TGTTTTATTA CTTGGTTGAG TACTTTTTCA CTCGTTAAAA

CDS9241_CDS8830R1 601 AGTTTTGAGA ATATTTTATA TTTTTGTTCA TGTAATCACT CCTTCTTAAT

CDS9241_CDS8830R1 651 TACAAATTTT TAGCATCTAA TTTAACTTCA ATTCCTATTA TACAAAATTT

CDS9241_CDS8830R1 701 TAAGATACAA ATCAAACAAA TTTTGGGCCC GGGGCGCGCC TGATACCTCG

CDS9241_CDS8830R1 751 CCAGATACTG CTTTACCATT AGCAAAAATC TAGTGGACAG CTTAAAAGGA

CDS9241_CDS8830R1 801 AGAATGAAAA ACAAAGATTA CTCGGAAATG GTTGTTCTGG GTGATTTTTG

CDS9241_CDS8830R1 851 CTATAATAGA AAGGTCTAAA GGATAAAAGA AAGGATTTAT ATGAAAACAT

CDS9241_CDS8830R1 901 TAGAAAAAAA ACTGGCAGAA GACTTTAAGA TCGTCTTTTC TGACAAGGAA

CDS9241_CDS8830R1 951 TTATTGGAAA CTGCCTTTAC TCATACTAGT TATGCTAATG AGCATCGCCT

CDS9241_CDS8830R1 1001 CCTAAACATT TCACATAACG AGCGCTTGGA ATTTTTAGGA GACGCTGTCT

CDS9241_CDS8830R1 1051 GCAGTTACGA TTTCACATTA TCTTTTTGAC AAATACCCTC AAAAGCTGAA

CDS9241_CDS8830R1 1101 GGTGA
