# Supplementary material for: Modulation of Biofilm Exopolysaccharides by the Streptococcus mutans vicX Gene
Source: Front Microbiol. 2015 Dec 21;6:1432. doi: 10.3389/fmicb.2015.01432 (PMC4685068; doi:10.3389/fmicb.2015.01432)
Supplement: Supplementary file 7 [file DataSheet1.ZIP › SmuvicX_DNA_sequencing/SmuvicX_DNA_sequencing_file5.pdf]

CDS9241_CDS8908D 1 CTTTATTTGG GCCATAGTGA GGAAGGCGAA GGGTCGACCT TTACCATCGT

CDS9241_CDS8908D 51 TTTACCTTAT GAAAACGATA ATGATGCAAT TGATGAATGG GAAGAAGATG

CDS9241_CDS8908D 101 AAGACGAATC ATGACAGAAA CAGGTTTTAG ATACAGCATT CTGGCTTCTG

CDS9241_CDS8908D 151 GTTCAAGTGG CAATAGTTTT TATTTGGAAA CTCCTCAAAA GAGAATTTTA

CDS9241_CDS8908D 201 ATTGACGCAG GCCTCTCAGG TAAGAAAATT AGTCAATTAA TGGCAGAAAT

CDS9241_CDS8908D 251 AGACCGAAAA GTGGCCGGCC AGCAGCAGCG ACTCATAGAA TTATTTCCTC

CDS9241_CDS8908D 301 CCGTTAAATA ATAGATAACT ATTAAAAATA GACAATACTT GCTCATAAGT

CDS9241_CDS8908D 351 AACGGTACTT AAATTGTTTA CTTTGGCGTG TTTCATTGCT TGATGGAACT

CDS9241_CDS8908D 401 GATTTTTAGT AAACAGTTGA CGATATTCTC GATTGACCCA TTTTGAAACA

CDS9241_CDS8908D 451 AAGTACGTAT ATAGCTTCCA ATATTTATCT GGAACATCTG TGGTATGGCG

CDS9241_CDS8908D 501 GGTAAGTTTT ATTAAGACAC TGTTTACTTT TGGTTTAGGA TGAAAGCATT

CDS9241_CDS8908D 551 CCGCTGGCAG CTTAAGCAAT TGCTGAATCG AGACTTGAGT GTGCAAGAGC

CDS9241_CDS8908D 601 AACCCTAGTG TTCGGTGAAT ATCCAAGGTA CGCTTGTAGA ATCCTTCTTC

CDS9241_CDS8908D 651 AACAATCAGA TAGATGTCAG ACGCATGGCT TTCAAAAACC ACTTTTTTAA

CDS9241_CDS8908D 701 TAATTTGTGT GCTTAAATGG TAAGGAATAC TCCCAACAAT TTTATACCTC

CDS9241_CDS8908D 751 TGTTTGTTAG GGAATTGAGA CTGTAGAATA TCTTGGTGAA TTAAAGTGAC

CDS9241_CDS8908D 801 ACGAGTATTC AGTTTTAATT TTTCTGACGA TAAGTTGAAT AGATGACTGT

CDS9241_CDS8908D 851 CTAATTCAAT AGACGTTACC TGTTTACTTA TTTTAGCCAG TTTCGTCGTT

CDS9241_CDS8908D 901 AAATGCCCTT TACCTGTTCC AATTTCGTAA CGGTATCGGT TTCTTTTAAA

CDS9241_CDS8908D 951 TTCAATTGTT TATTACTTGG TTGAGTACTT TTTCACTCGT TAAAAAGTTT

CDS9241_CDS8908D 1001 TGAGATATTT ATATTTTGTC ATGTAATCAC TCTCTACTAC AATTTTAGCA

CDS9241_CDS8908D 1051 TCTATTTACT TCATTCCTAT TATACAAATT TTAAGAATAC AAATCGAC
