# Supplementary material for: Modulation of Biofilm Exopolysaccharides by the Streptococcus mutans vicX Gene
Source: Front Microbiol. 2015 Dec 21;6:1432. doi: 10.3389/fmicb.2015.01432 (PMC4685068; doi:10.3389/fmicb.2015.01432)
Supplement: Supplementary file 7 [file DataSheet1.ZIP › SmuvicX_DNA_sequencing/SmuvicX_DNA_sequencing_file6.pdf]

CDS9241_CDS8830R 1 CTTATACGTA CTTTGTTTCA AATGGGTCAA TCGAGAATAT CGTCAACTGT

CDS9241_CDS8830R 51 TTACTAAAAA TCAGTTCCAT CAAGCAATGA AACACGCCAA AGTAAACAAT

CDS9241_CDS8830R 101 TTAAGTACCG TTACTTATGA GCAAGTATTG TCTATTTTTA ATAGTTATCT

CDS9241_CDS8830R 151 ATTATTTAAC GGGAGGAAAT AATTCTATGA GTCGCTGCTG CTGGCCGGCC

CDS9241_CDS8830R 201 ACTTTTCGGT CTATTTCTGC CATTAATTGA CTAATTTTCT TACCTGAGAG

CDS9241_CDS8830R 251 GCCTGCGTCA ATTAAAATTC TCTTTTGAGG AGTTTCCAAA TAAAAACTAT

CDS9241_CDS8830R 301 TGCCACTTGA ACCAGAAGCC AGAATGCTGT ATCTAAAACC TGTTTCTGTC

CDS9241_CDS8830R 351 ATGATTCGTC TTCATCTTCT TCCCATTCAT CAATTGCATC ATTATCGTTT

CDS9241_CDS8830R 401 TCATAAGGTA AAACGATGGT AAAGGTCGAC CCTTCGCCTT CCTCACTATT

CDS9241_CDS8830R 451 GGCCCAAATA AAGCCTTTAT GCTGTTTGAC AATTTCTTTG GCGATTGCTA

CDS9241_CDS8830R 501 ATCCTAAACC AGTCCCACCT TGAGCACGGC TTCTTGCCTT ATCAACACGA

CDS9241_CDS8830R 551 TAAAAACGGT CAAAAATAAG AGGGAGATCC TTCTTAGGAA ACCCCAAGCC

CDS9241_CDS8830R 601 TAA
